# Buddleoside-rich *Chrysanthemum indicum* L. extract modulates macrophage-mediated inflammation to prevent metabolic syndrome induced by unhealthy diet

**DOI:** 10.1186/s12906-024-04583-2

**Published:** 2024-08-23

**Authors:** Yiqing Zhou, Jie Su, Yingjie Dong, Ziwen He, Yajun Wang, Suhong Chen, Guiyuan Lv

**Affiliations:** 1 College of Pharmaceutical Science, Zhejiang Chinese Medical University, No. 548, Binwen Road, Binjiang District, Hangzhou, Zhejiang 310053 China; 2 Collaborative Innovation Center of Yangtze River Delta Region Green Pharmaceuticals, Zhejiang University of Technology, No. 18, Chaowang Road, Xiacheng District, Hangzhou, Zhejiang 310014 China; 3 Zhejiang Provincial Key Laboratory of TCM for Innovative R & D and Digital Intelligent Manufacturing of TCM Great Health Products, Huzhou, 313200 China

**Keywords:** Metabolic syndrome, Buddleoside-rich *Chrysanthemum indicum* L. extract, Insulin resistance, Inflammation

## Abstract

**Background:**

Metabolic syndrome (MetS) is a precursor to the development of many diseases (atherosclerosis, diabetes, etc.). It is marked by disruptions in glucose and lipid metabolism, along with hypertension. Numerous types of risk factors contribute to the development of the MetS, inflammation and insulin resistance are present throughout the metabolic abnormalities. *Chrysanthemum indicum* L. is a traditional Chinese plant used for both tea and medicine, known for its high content of total flavonoids, which are important secondary metabolites. Our research led to the extraction of a Buddleoside-Rich *Chrysanthemum indicum* L. extract (BUDE) which has demonstrated anti-inflammatory properties. Nonetheless, the specific role and mechanism of BUDE in preventing MetS remain unclear.

**Methods:**

The study initially evaluated the role of BUDE in preventing MetS. Subsequently, it investigated the anti-inflammatory properties of BUDE in the liver and pancreas in response to unhealthy diets. It then examined the level of insulin resistance and pancreatic β-cell function induced by inflammation. Additionally, an lipopolysaccharide (LPS)-induced macrophage inflammation model was used to further investigate the ameliorative effects of BUDE in inflammation.

**Results:**

BUDE has hypotensive, hypoglycemic and hypolipidemic effects. It can also resolve the imbalance between macrophage subpopulations, impede the triggering of the NF-κB signaling pathway, reduce the secretion of inflammatory mediators, ameliorate insulin resistance, and safeguard organs such as the liver and pancreas from inflammatory damage. These effects collectively contribute to preventing the development of MetS.

**Discussion:**

BUDE has the ability to modulate macrophage-mediated inflammation, leading to improved insulin resistance. Additionally, it delivers antihypertensive, hypoglycemic, and hypolipidemic effects, offering a potential for preventing MetS.

**Supplementary Information:**

The online version contains supplementary material available at 10.1186/s12906-024-04583-2.

## Background

Absolute burden of metabolic syndrome (MetS) is on the rise globally [1], the implementation of effective interventions to slow and reverse the current epidemic of the MetS and thereby reduce the associated morbidity and mortality should become an escalating public health issue worldwide [2]. The syndrome’s pathogenesis results from the aggregation of multiple risk factors, with unhealthy diet and lifestyle being significant contributors [3]. Its clinical manifestations are composed of aggregates of hyperlipidemia, hyperglycemia, and hypertension. MetS induces significant organ damage and amplifies the associated cardiovascular risks, increasing the risk of disease and death [4]. Given the wide-ranging nature of the effects of MetS, coupled with the lack of a controllable ability to stay healthy living a stressful life and the absence of specific medications for the prevention of MetS, there is an urgent quest for a medication that can prevent MetS in order to prevent the absolute burden that MetS induces.

The MetS is closely associated with inflammation. Nutrient and metabolic overload can trigger inflammation and lead to dysregulation of macrophage polarization, causing inflammatory macrophage infiltration in metabolic tissues and organs [5]. In a state of chronic overconsumption of fats, sugar, and alcohol, the body tends to develop a pro-inflammatory macrophage phenotype [6]. This leads to elevated expression of inflammatory cytokines, resulting in systemic manifestations of hypo- and brady-inflammation. This inflammation results in reverse fat storage and severe inflammatory damage in organs like the liver [7], while also impairing insulin signaling and reducing tissue sensitivity, ultimately leading to insulin resistance. Poor diet will cause an increase in blood glucose, a small fluctuation in blood glucose can trigger a huge change in insulin-producing cells thus causing an elevation in insulin levels, causing hyperinsulinemia to be the primary symptom of insulin resistance in its early phases [8]. Excessive insulin may even affect vascular tone and sympathetic activity, leading to disruptions in blood pressure regulation. The most sensitive organ, the liver plays a critical role in determining metabolic disorders [9], experiencing insulin damage that occurs more rapidly than other organs; accordingly, hepatic insulin resistance is the main event leading to the subsequent development of insulin resistance in peripheral tissues [10]. It is worth noting, insulin resistance in pancreatic islet cells cannot be ignored [11]. However, that the analysis of mechanisms for correcting metabolic abnormalities using insulin resistance failed to evaluate some non-insulin-sensitive tissues. In order to fully simulate its MetS, this study replicated the model using a high-fat, high-sugar combination with alcohol consumption, in which, in addition to the metabolic burden brought about by the excess of nutritional factors, there is a metabolic disorder amplified by the catalytic effect brought about by the risk element of alcohol.

*Chrysanthemum indicum* L., a common health food, is widely used to make teas. It is a medicinal plant and recent studies have shown that possesses a variety of pharmacological properties, including hepatoprotective [12, 13], immunomodulatory [14], anti-inflammatory [15–17], and antihypertensive properties. Notably, no reports of associated negative effects exist. Flavonoids, terpenoids, phenylpropanoids, and phenolic acids are among its chemical ingredients [18]. The content of total secondary metabolites flavonoids in *Chrysanthemum indicum* L. is the highest, playing an important role. Studies have suggested that the flavonoid composition can treat inflammation, reduce inflammatory bowel disease [19]. Furthermore, it has been found to be effective in improve insulin resistance and lower triglyceride and cholesterol levels [20, 21]. Our previous studies extracted Buddleoside-Rich *Chrysanthemum indicum* L. extract (BUDE) from *Chrysanthemum indicum* L. and found it to have therapeutic effects on blood pressure [22, 23], as well as protection to vascular endothelial cells from inflammatory damage [24], among other benefits. These ranges of efficacy triggered us to think about whether BUDE could prevent MetS. However, to understand its value in preventing MetS and its underlying mechanisms, more research is necessary. Given the centrality of insulin resistance in metabolic disorders [25], inflammation and insulin resistance are pervasive in their metabolic abnormalities [26]. Thus, our study explores the potential of BUDE in preventing MetS by addressing inflammation and insulin resistance to correct metabolic abnormalities.

This study looked into the effects of BUDE on rats with MetS, which are brought on by a diet heavy in fat and sugar with overdose of alcohol consumption. The results obtained indicate that BUDE’s inhibition of the NF-κB signaling pathway may ameliorate systemic chronic inflammation. The protective effect of BUDE helps to reduce diet-induced inflammation of the liver and pancreas, improve insulin resistance, correct metabolic abnormalities, and prevent MetS.

## Materials and methods

### Experimental animals

The Experimental Animal Center of Zhejiang Academy of Medical Sciences (License No. SYXK (Zhe) 2019-0001) supplied six-week-old male SD rats for the study. The animal experiments were carried out in compliance with the National Research Council’s Guide for the Care and Use of Laboratory Animals. The welfare of animals and the experimental procedures were approved by the Institutional Animal Care and Use Committee of Zhejiang Chinese Medical University (Hangzhou, China) (ZSLL-2017-040) in 2017. All rats were acclimatized and fed for one week, their basal blood pressure was measured and randomized into 4 groups according to their basal blood pressure, which were the normal group (NG, *n* = 10), the model group (MG, *n* = 10), the BUDE low-dose group (BUDE-L, 75 mg·kg^− 1^, *n* = 10), and the BUDE high-dose group (BUDE-H, 150 mg·kg^− 1^, *n* = 10). All rats except normal rats were fed high-fat chow (15% lard, 0.8% cholesterol, 20% sucrose, 0.2% sodium cholate, and 64% regular chow; protein: fat: carbohydrate = 19:37:44), as well as compound gradient drinking. During the modeling process, the rats in the drug administration group were given a daily dose of the designated test drug, while the normal and model groups were given water gavages for a duration of six weeks. All rats were then euthanized with an intraperitoneal injection of 0.2 g/mL urethane at a volume of 0.6 mL/100 g, ensuring the animals were relieved of stress and discomfort while maintaining a stable physiological state.

### Cells and cell culture

Mouse monocyte macrophage leukemia cells (RAW264.7), derived from laboratory reserve, were cultured in DMEM (Gibco, USA) medium supplemented with 100 U/mL streptomycin-penicillin (Biosharp, China) and 10% fetal bovine serum (Four Seasons, China) at 37 °C in 5% CO_2_. Cells in logarithmic growth phase were then inoculated in 6-well plates with a cell suspension of 1 × 10^6^ cells/well (2 mL per well). After 24 h of plate laying, a blank control group, a model control group, and three BUDE groups with different concentrations (25, 15, and 5 µM) were established. One hour after drug administration, while the other groups were given lipopolysaccharide (LPS) at 200 ng/mL to simulate the inflammatory response, the blank control group was given the same amount of baseline media. The drug action was continued for 6 hours for further subsequent experiments.

### Tested drugs

*Chrysanthemum indicum* L. was acquired from Zhejiang Chinese Medical University, Traditional Chinese Medicine Decoction Pieces Co., Ltd. (Hangzhou, Zhejiang, China, LOT: 2,001,043). Its identification can be referenced in the 2020 Chinese Pharmacopoeia. The drug used in this study was derived from BUDE that had been extracted in a previous study. In the previous study, the results of HPLC analyses demonstrated that BUDE contained 69.62 ± 0.78% of BUD [23]. The required BUDE for rats were obtained by weighing the dry powder of BUDE and configuring it with pure water. The dose for rats in this study was derived from the adult dosage in the Chinese Pharmacopoeia and obtained by converting the surface area of the human body. The specific dose was mainly referred to the results of previous studies. For cells, BUDE prepared by dissolving BUDE powder in DMSO (Shanghai yuanye, China).

### UPLC-Q-TOF/MS analysis of BUDE

BUDE sample precision weighing 10.0 mg, add 70% methanol 1.0 mL, room temperature ultrasonic extraction twice, each time 30 min, 12,000×g centrifugation for 10 min, take the supernatant filtered by 0.2 μm filter membrane, spare. Then weigh the appropriate amount of control product, add methanol to dissolve ultrasonication, 4℃ stored away from light, spare. The analytes were analyzed by ultra-high performance liquid chromatography quadrupole tandem time of flight mass spectrometry (UPLC-Q-TOF/MS) (Waters SYNAPT G2-Si). The chromatographic column was CORTECS^®^UPLC^®^T3 (2.1 × 100 mm, 1.6 μm); the mobile phases were 0.1% formic acid in water and pure acetonitrile; the gradient elution: 0–2 min, 5% acetonitrile; 2–32 min, 5-100% acetonitrile; 32–33 min, 100% acetonitrile; 33.5 min, 5% acetonitrile; 33.5–35 min, 5% acetonitrile flow rate 0.3 mL/min, injection volume 2 uL, column temperature 35℃, sample chamber temperature 10℃.

Ion source parameters for mass spectrometry: capillary voltage: positive: 3.0 kV; negative: 2.5 kV; sample cone-well voltage: 40 V; source offset voltage: 80 V; ion source temperature: 120℃; desolventization temperature: positive 500℃, negative 400℃; desolventization flow rate: positive 1000℃, negative 800℃; nebulizing gas pressure: 6.5 Bar.

Mass spectrometry methods: electrospray ESI ion source, positive and negative ion modes were scanned separately, MSE continue full scan mode, scanning time 0.2 s, scanning range m/z 50-1200. Collision energy was used in MSE, the low collision energy was 6 V, and the high collision energy was 15–45 V. Sodium formate was used for the mass spectrometry calibration, and the leucine enkephalins (positive ion mode m/z 556.2771, negative ion mode m/z 554.2615) were used for real-time mass calibration.

### Blood pressure detection

An intelligent noninvasive blood pressure monitor (BP-2010AUL) was utilized to take the rats’ blood pressure once a week. The process began by setting the temperature of the blood pressure chamber to 26 ± 1 °C. The rats to be tested were then placed in the chamber and allowed to acclimatize for at least 15 min. Following this, the rats were immobilized in appropriately sized cages equipped with a thermostat, and the temperature was adjusted to 40 °C. Eventually, the rats’ tails were secured through the holes in the cage and inserted into the system’s pulse sensor. After the signal wave had steadied, the device pressurized automatically to gauge and document diastolic blood pressure (DBP), systolic blood pressure (SBP), and mean arterial pressure (MBP).

### Detection of serum glycolipid level

After the rats were fasted for 12 h, but still had access to water or alcohol, blood was obtained and placed in a 37℃-water bath for 30 min. After that, it was centrifuged at 3500 r·min^− 1^ for 10 min to separate the serum. The serum was then tested for fasting blood glucose (FBG), triglycerides (TG), total cholesterol (TC), low-density lipoprotein cholesterol (LDL-c), and high-density lipoprotein cholesterol (HDL-c) using a reagent kit (Medicalsystem Biotechnology Co., Ltd. China) with a Fully automated biochemistry analyzer (TBA-40FR).

### Glucose tolerance test

Rats fasted on food but not water/alcohol for 12 h and then their tail tips were pricked with a blood collection needle. The initial blood droplet was removed using a sterile gauze, and the subsequent droplet was then applied to the test area of the glucose test strip. Subsequently, glucose (2.0 g/kg body mass) was administered by gavage, and the glucose concentration of the rats was measured by a glucometer (Sinocare, China) at 30, 60, and 120 min following the administration of glucose. Moreover, the trapezoidal rule was utilized to calculate the area under the blood glucose curve (AUC) in order to more accurately evaluate glucose tolerance and assess β-cell function.

### Enzyme-linked immunosorbent assay (ELISA)

Appropriate amount of serum was taken and routinely thawed to determine the levels of serum Interleukin-6 (IL-6), lipopolysaccharide (LPS) and Fasting Insulin (FINS) by ELISA, and the supernatants of cultured RAW264.7 cells were collected to determine the levels of interleukin-1β (IL-1β) and IL-10 by ELISA. The experimental procedure was performed in strict compliance with the instruction manual of the kit (Jiangsu Meimian Industry Co., Ltd). The insulin resistance index (IRI) and insulin sensitivity index (ISI) were calculated from the corresponding data. IRI = FBG×FINS/22.5 and ISI = ln(1/(FBG×FINS)).

### Histological analysis

The pancreas and liver of rats were washed using phosphate-buffered saline (PBS) and then preserved in 4% paraformaldehyde. Afterwards, the tissues were routinely dehydrated, paraffin-embedded, and were cut into 4 μm thick sections. Hematoxylin and eosin staining (H&E) was performed according to standard procedures to visualize morphologic alterations of the liver and pancreatic tissues, which were then photographed under a microscope (MF43-N, China).

### Histological evaluations and immunohistochemistry (IHC)

For immunohistochemistry, rat liver and pancreas tissue sections were dewaxed and hydrated, repaired with sodium citrate antigen repair solution, blocked with endogenous peroxidase (ZSGB-BIO, China) according to the instructions, inhibited for ten min using an immunostaining blocking solution. The primary antibody IL-6 (R1412-2, HUABIO) or IL-1β (66737-1-lg, Proteintech) dilution (1:500) were added dropwise and stored in the refrigerator at 4℃ overnight. Subsequently, the corresponding secondary antibody was added dropwise and incubated in an oven at 37 °C for 30 min, followed by DAB color development, hematoxylin staining of the nucleus, and finally dehydrated transparent sealing solution. The expression of liver tissue as well as pancreatic IL-6 and IL-1β were observed under the microscope.

### Immunofluorescence (IF)

For pancreatic immunofluorescence, the rat pancreatic tissue sections were deparaffinized, repaired with sodium citrate antigen repair solution, closed with immunostaining blocking solution for 10 min. The sections were then added with primary antibody IRS2 (R382966, Zenbio, 1:200), INS Monoclonal antibody (66198-1-Ig, Proteintech, 1:250) and Glucagon polyclonal antibody (15954-1-AP, Proteintech, 1:500) mixture overnight at 4℃. After washing with PBS three times, rabbit secondary antibody (SA00013-4, Proteintech) was added dropwise at 37℃ for 30 min. For fluorescent double-stained sections, the procedure was repeated with mouse secondary antibody (HA1128, HUABIO) after three rinses with PBS. Finally, antifluorescence quencher containing DAPI was added, a clean coverslip was placed over the antifluorescence quencher, the position of the coverslip was adjusted, and after 10 min, the islet cells were observed and photographed under a microscope (Zeiss SteREO Discovery. V20, Germany).

For RAW264.7 cells immunofluorescence, LPS-induced RAW264.7 cells were washed with PBS at a density of 2×l0^5^ per well in 12-well dishes with cell slides. Next, the cells were immobilized by exposing them to 4% paraformaldehyde for a duration of 20 min at ambient temperature. Following three PBS washes, the cells were permeabilized with membrane-breaking working solution (TritonX-100, 0.1%) for 20 min at ambient temperature. 5% bovine serum albumin (BSA) was blocked at room temperature for 2 h. The cell slides were treated with prepared antibody against NF-κB (ET1603-12, HUABIO) overnight at 4 °C in a humidified box. After being rinsed with PBS, the cells were subjected to treatment with the identifying secondary antibody to the primary antibody and left to stand at room temperature for an hour. Then antifluorescence-quenched seals containing DAPI were added, and the cells were left to incubate at room temperature for 10 min, shielded from light, and stored at 4 °C. All cells were imaged by laser confocal microscopy (Carl Zeiss, Germany).

### Liver glycogen content assay

The glycogen content in rat liver was assessed using a commercial kit supplied by Nanjing Jiancheng Bioengineering Institute (Nanjing, China). In summary, the liver samples were thawed from a -80 °C refrigerator, promptly weighed, homogenized with three times the volume of alkaline solution, and then hydrolyzed by heating for 20 min in a boiling water bath. The cooled hydrolysate was diluted with distilled water to achieve the 5% concentration necessary for determining liver glycogen. Following this, the liver glycogen content was measured at 620 nm.

### Quantitative real-time polymerase chain reaction PCR (qRT-PCR)

Animal samples consisted of rat liver tissues, collected in appropriate amounts, while cell samples were obtained from RAW264.7 cells after differentiation induction with LPS and drug administration as described above. Both RAW264.7 cells and rat liver tissues have their whole RNA extracted using the Steady Pure Rapid RNA Extraction Kit (Accurate Biology). The RNA concentration of each sample was then determined using an ultra-micro spectrophotometer. Subsequently, single-stranded cDNA was created by reverse transcribing 1 µg of total RNA following the instructions of the MonScript™ RTIII Super Mix with dsDNase (Two-Step) (Monad). The resulting cDNA was then used for quantitative PCR in a real-time fluorescence quantitative PCR instrument (StepOnePlus) with MonAmp™ SYBR ^®^ Green qPCR Mix (High ROX) (Monad). Semi-quantitative analysis was conducted using the ΔΔCt method, and finally the data were normalized using the RNA expression level of β-Actin. The gene sequences of each primer are detailed in the following Table 1.

**Table 1:** Primer sequence list

Gene	Genbank Accession	Primer	Sequence(5’to 3’)
Rat ACTB	NM_031144.3	Forward	5’-CTATCGGCAATGAGCGGTTC-3’
		Reverse	5’-CAACGTCACACTTCATGATGG-3’
Rat IRS1	NM_012969.2	Forward	5’-GAAACGCCACAGCTCTGCATC-3’
		Reverse	5’-GGTGCTGCTTAACATCCTTGACC-3’
Rat IRS2	NM_001168633.1	Forward	5’-TTCCAAGCGCCACAATTCGG-3’
		Reverse	5’-ATGGCTCATCACTTCCTCCC-3’
Rat GLUT2	NM_012879.2	Forward	5’-TCATCGCCCTCTGCTTCCAGT-3’
		Reverse	5’-GGCCGAACCACTCTTCTTCCG-3’
Mouse GAPDH	NM_001289726.2	Forward	5’-AGGTCGGTGTGAACGGATTTG-3’
		Reverse	5’-TGTAGACCATGTAGTTGAGGTCA-3’
Mouse IL-1β	NM_008361.4	Forward	5’-GCCACCTTTTGACAGTGATG − 3’
		Reverse	5’-AAGGTCCACGGGAAAGACAC-3’
Mouse IL-6	NM_001314054.1	Forward	5’-GGAGACTTCACAGAGGATAC-3’
		Reverse	5’-GCATCATCGTTGTTCATACA-3’

### Western blotting (WB)

The cells/tissues were lysed in a lysis solution containing protease and phosphatase inhibitors. Subsequently, proteins were separated via SDS-PAGE and then moved onto a polyvinylidene difluoride membrane. The membranes were then incubated in phosphate buffer solution containing 2.0% Tween (TBST) and 20% BSA for 2 h. Following this, the membranes were then treated with IκB (YT2419, Immunoway), p-IκB (YP1372, Immunoway), NF-κB (HA721307, HUABIO), p-NF-κB (db7996, Diagbio), and β-actin (YT0099, Immunoway) for an entire night at 4 °C. The following day, the membranes were washed in TBST and exposed to the appropriate secondary HRP-coupled antibody for 2 h at room temperature. Ultimately, a chemiluminescence detector and an enhanced chemiluminescence solution (ECL) were used to see the protein bands. The enhanced chemiluminescence solution was rinsed off with TBST, then the previously incubated antibody was eluted with stripping buffer (Solarbio, sw3022), rinsed again with TBST, and the primary antibody incubation and subsequent procedure described above was repeated.

### Statistics

Statistical analysis was conducted using SPSS 25.0 software. The data were presented as mean ± standard deviation (± s). Group comparisons were performed using one-way ANOVA and t-tests. Statistical significance was denoted by *P* < 0.05 and *P* < 0.01.

## Results

### Chemical composition of BUDE analyzed by UPLC-Q-TOF/MS

**Fig. 1:**
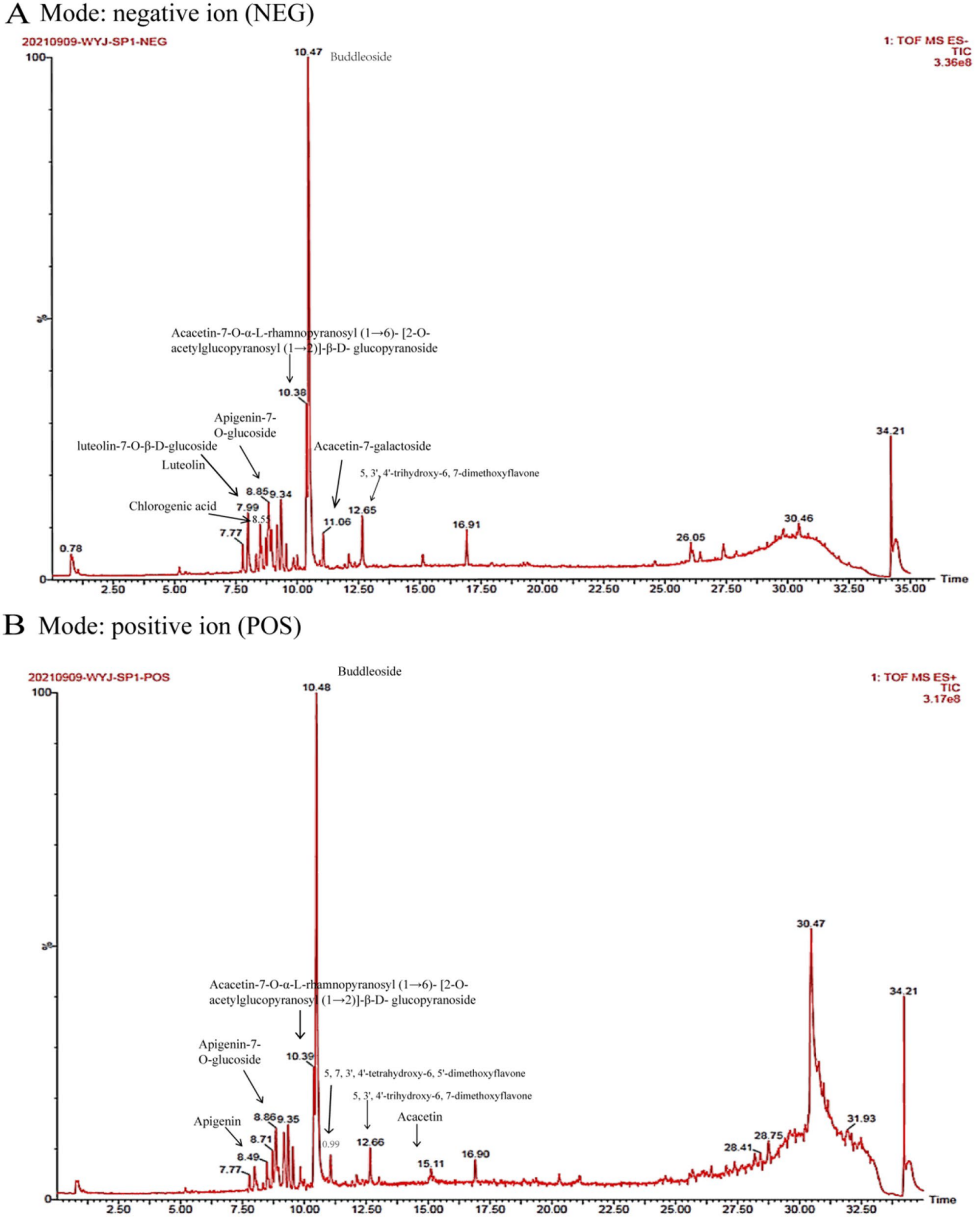
Ion chromatogram of Buddleoside-Rich *Chrysanthemum indicum* L. extract (BUDE) by UPLC-Q-TOF/MS (**A**) Negative ion mode. (**B**) Positive ion mode

The BUDE constituents were identified by analyzing the chromatographic retention time and mass spectrometry data, which were compared with reference compounds. In addition to BUDE, 10 other components were found in the sample. Figure 1 illustrates the positive and negative ion chromatograms of BUDE, while Table 2 summarizes the compound identification data.

**Table 2:** Identification of the compounds in BUDE

Number	t_*R*_(min)	Formula	Ionic mode	Measuremass (m/z)	MS^2^ Ions (m/z)	Identified compound
1	10.48	C_28_H_32_O_14_	[M + HCOO]^−^	637.1820	268.0400, 283.0636, 284.0665, 566.5280	Buddleoside
2	10.42	C_36_H_43_O_20_	[M + HCOO]^−^	841.2479	239.0362, 426.1780, 623.1659, 753.2280	Acacetin-7-O-α-L-rhamnopyranosyl(1$\rightarrow$6)-[2-O-acetylglucopyranosyl(1$\rightarrow$2)]-β-D-glucopyranoside
3	8.0	C_21_H_20_O_11_​	[M-H]^−^	447.0957	116.9289, 283.0269, 285.0421, 286.0454	luteolin-7-O-β-D-glucoside
4	8.81	C_21_H_20_O_10_​	[M-H]^−^	431.1005	268.0385, 269.0460	Apigenin-7-O-glucoside
5	11.06	C_22_H22O_10_​	[M + HCOO]^−^	491.1206	237.0917, 268.0382, 283.0617, 284.0651	Acacetin-7-galactoside
6	15.13	C_16_H_12_O_5_​	[M-H]^−^	283.0620	237.0918, 268.0379	Acacetin
7	7.99	C_15_H_10_O_6_​	[M-H]^−^	285.0421	133.0299, 116.9289, 283.0269	Luteolin
8	8.49	C_15_H_10_O_5_​	[M-H]^−^	269.0469	98.9496	Apigenin
9	8.55	C_16_H_18_O_9_​	[M-H]^−^	353.0887	135.0455, 191.0565	Chlorogenic acid
10	10.99	C_17_H_14_O_8_​	[M-H]^−^	345.0624	315.0157, 330.0382	5,7,3’,4’-tetrahydroxy-6,5’-dimethoxyflavone
11	12.53	C_17_H_14_O_7_​	[M-H]^−^	329.0672	221.8442, 283.0613	5, 3’, 4’-trihydroxy-6, 7-dimethoxyflavone

### BUDE can lower blood pressure and regulate lipid balance

**Fig. 2:**
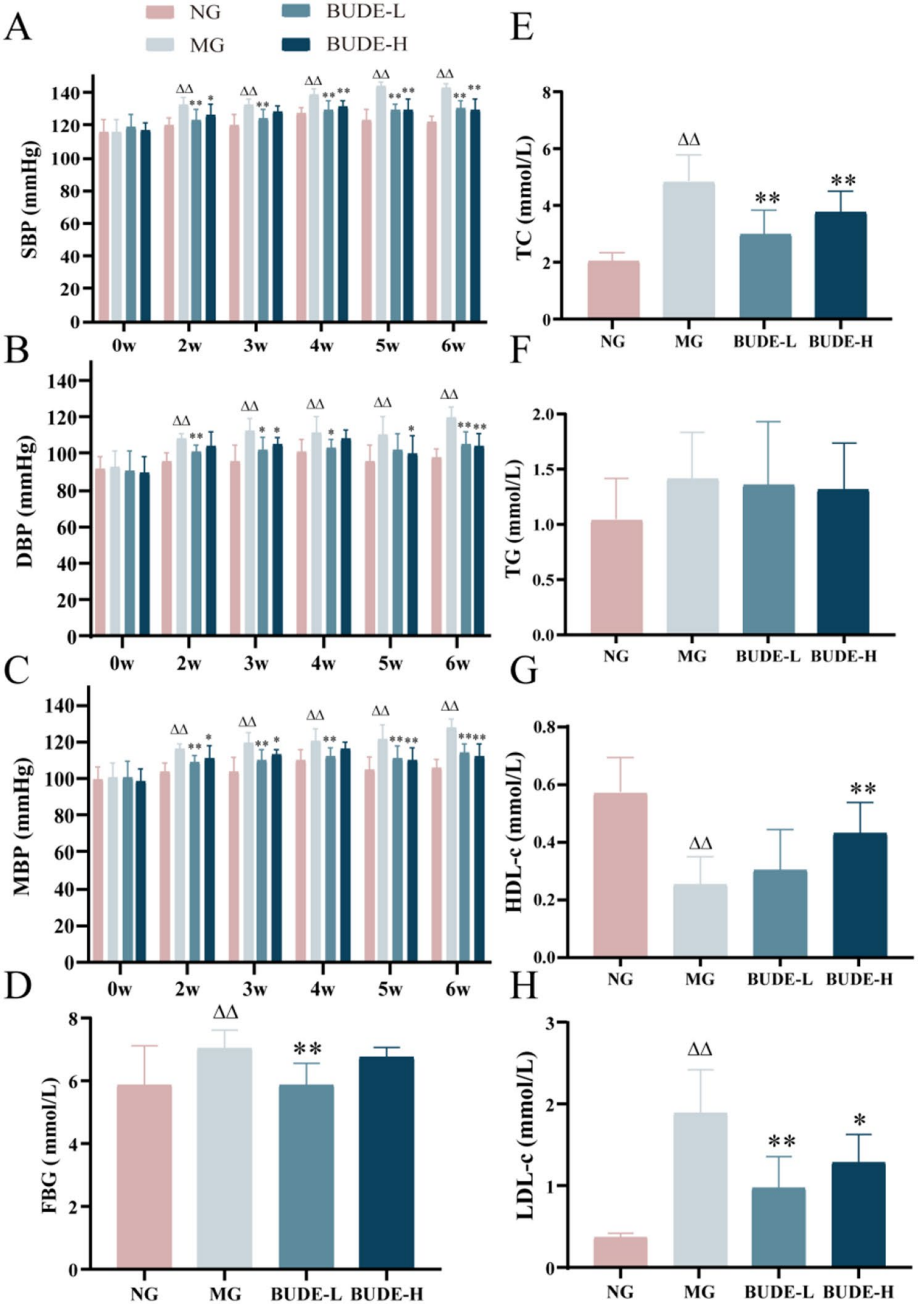
Effects of BUDE on blood pressure and glucolipid metabolism in model rats. (**A**-**C**) Changes in systolic blood pressure (SBP), diastolic blood pressure (DBP) and mean blood pressure (MBP). (*n* = 10). (**D**) Fasting blood glucose (FBG). (*n* = 10). (**E**-**H**) Changes in serum total cholesterol (TC), triglycerides (TG), high-density lipoprotein cholesterol (HDL-c) and low-density lipoprotein cholesterol (LDL-c). (*n* = 9–10). Compared with the normal group, ^▲^*P <* 0.05, ^▲▲^*P* < 0.01; compared with the model group, *P < 0.05, **P < 0.01. NG: normal control group; MG: model control group; BUDE-L: BUDE low dose (75 mg·kg^− 1^) group; BUDE-H: BUDE high dose (150 mg·kg^− 1^) group

Weekly during the 2–6 weeks of modeling/administration, rats in the model group exhibited notable elevated SBP, DBP, and MBP compared to the normal group (*P* < 0.01). Conversely, rats given BUDEs showed a notable decrease in SBP, DBP, and MBP relative to the model group (*P* < 0.01, 0.05) (Fig. 2A-C). Notably, after 5 weeks of modeling, SBP of rats in the model group exceeded 140 mmHg. Furthermore, the model group showed large reductions in HDL-c and significant rises in TC, LDL-c, and FBG with respect with the normal group *(P* < 0.01). Conversely, rats treated with BUDE-L exhibited substantial decreases in TC, LDL-c, and FBG as opposed to the model group (*P* < 0.01), rats administered with BUDE-H exhibited significant reductions in TC, LDL-c as well as substantial increases in HDL-c when matched to the model group (*P* < 0.01) (Fig. 2D-H). This study successfully established a MetS model and demonstrated that BUDE intervention effectively improved glucose and lipid metabolism disorders caused by excessive consumption of high sugar and high fat diet and too much alcohol, without significantly increasing blood pressure.

### BUDE Reduce Inflammation and inflammatory Injury

**Fig. 3:**
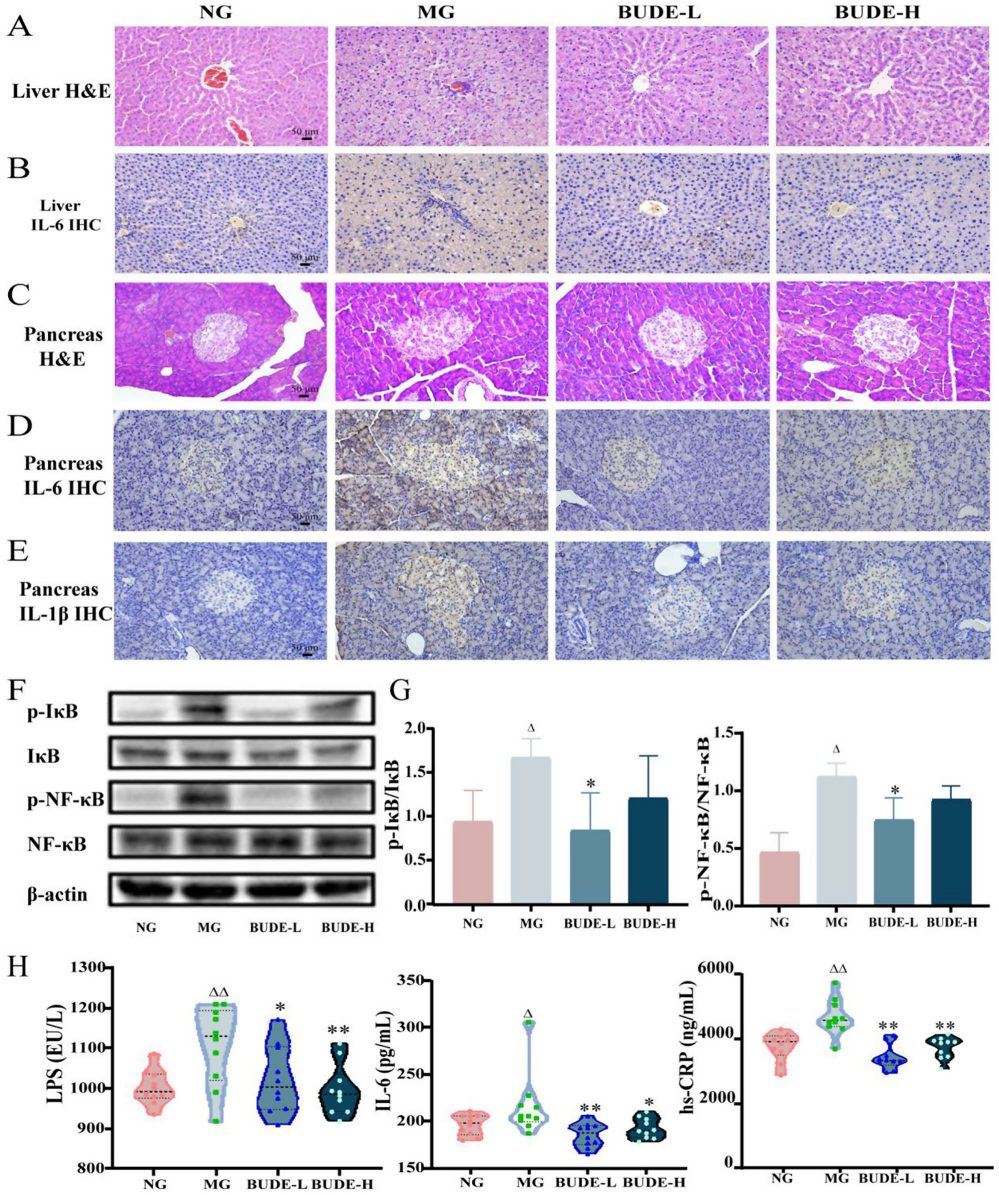
Effects of BUDE on inflammatory injury in model rats. (**A**) Representative graph of hematoxylin and eosin staining (H&E) of the liver (200×). (*n* = 3). (**B**) Representative graph of liver Interleukin-6 (IL-6) immunohistochemistry (IHC) (200×). (*n* = 3). (**C**) Representative graph of H&E of pancreas (200×). (*n* = 3). (**D**-**E**) Representative graphs of pancreatic IL-6 and Interleukin-1β (IL-1β) IHC (200×). (*n* = 3). (**F**) Representative graph of pancreatic NF-κB pathway-associated protein blotting. (**G**) Relative expression levels of p-NF-κB, p-IκB proteins. (*n* = 3). (**H**) Levels of serum lipopolysaccharide (LPS), IL-6, and high-sensitivity C-reactive protein (hs-CRP). (*n* = 10). Compared with the normal group, ^▲^*P <* 0.05, ^▲▲^*P <* 0.01; compared with the model group, *P < 0.05, **P < 0.01. NG: normal control group; MG: model control group; BUDE-L: BUDE low dose (75 mg·kg^− 1^) group; BUDE-H: BUDE high dose (150 mg·kg^− 1^) group

The results of HE staining revealed distinct differences between the model and normal groups. In the normal group, hepatocytes exhibited intact nuclei, clear cytoplasm, and well-defined hepatic cords, while the model group displayed vacuolated hepatocytes with missing nuclei, diffuse steatosis, and inflammatory cell aggregates. However, rats in the BUDE groups showed reduced liver lesions, with a significant decrease in vacuolization of hepatocytes, reduced inflammatory cell infiltration (Fig. 3A), and decreased hepatic IL-6 expression (Fig. 3B) compared to the model group. Similarly, in the normal group, pancreatic islet cells exhibited normal morphology with clearly visible nuclei and well-defined borders, while the model group showed irregular pancreatic islets with unclear borders and inflammatory cell aggregates (Fig. 3C). Administration of the drug improved pancreatic lesions to some extent, with reduced levels of IL-6 and IL-1β in the pancreas (Fig. 3D-E). Additionally, ELISA data showed that the model group had markedly higher amounts of inflammation-related components than the normal group did, while BUDE groups were able to significantly lower the levels of serum high-sensitivity C-reactive protein (hs-CRP) and IL-6 (*P* < 0.01, 0.05), as well as the levels of LPS (*P* < 0.01, 0.05), which promotes the release of various pro-inflammatory factors (Fig. 3H). WB results showed that BUDE-L remarkably reduced the expression of phosphorylated IκB and phosphorylated NF-κB (*P* < 0.05) (Fig. 3F-G). These findings suggested that BUDE has the potential to block the activation of NF-κB signaling pathway to mitigate liver and pancreatic inflammation and minimize the harm caused by “overindulge in fatty, sweet and thick flavors” in rats with MetS.

### BUDE improve insulin resistance and pancreatic β-cell function

**Fig. 4:**
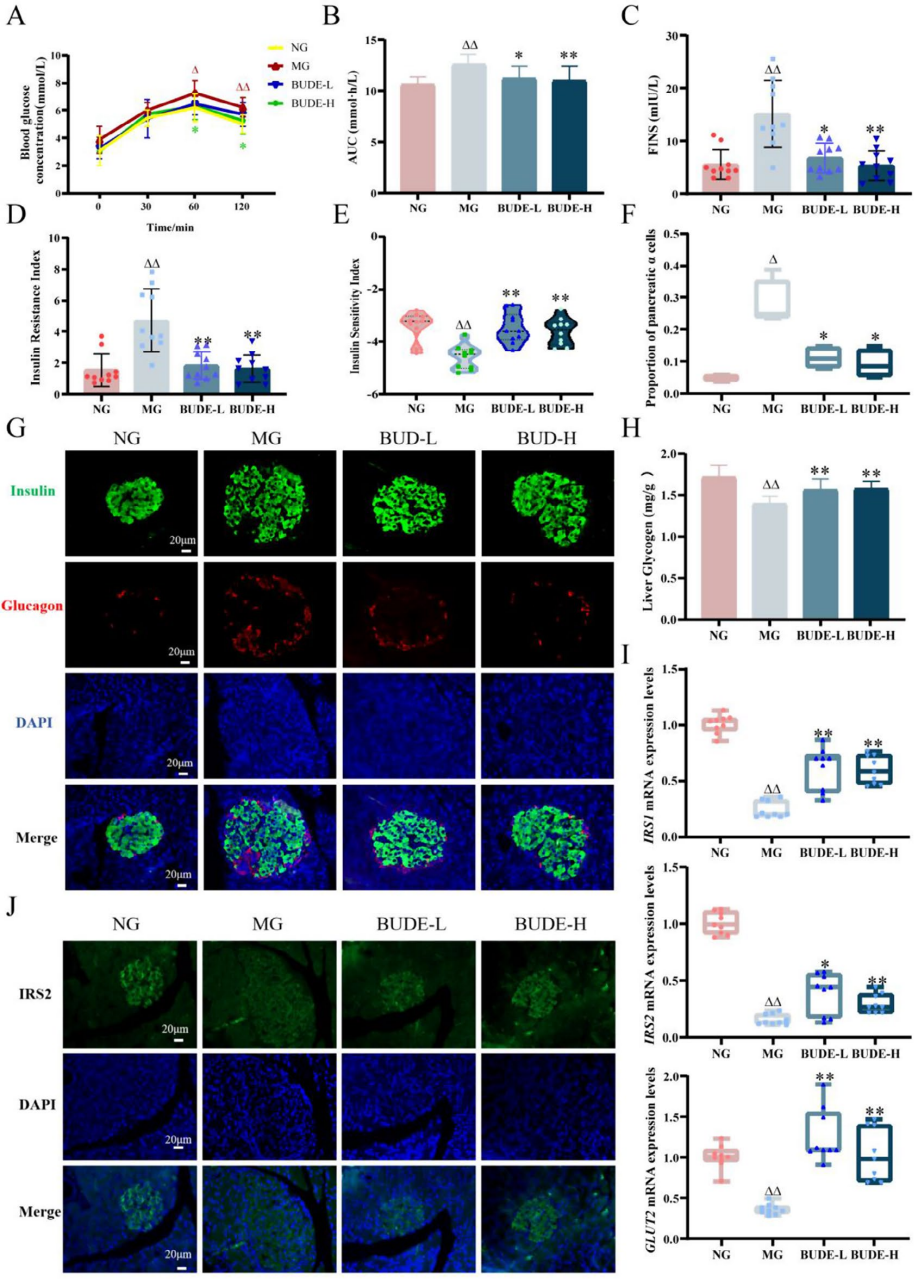
Effect of BUDE on insulin resistance in model rats. (**A**, **B**) Glucose tolerance test and area under the blood glucose curve. (*n* = 10). (**C**) Serum fasting insulin levels. (*n* = 10). (**D**-**E**) Insulin resistance index and insulin sensitivity index. (*n* = 10). (**F**) Representative image of pancreatic immunofluorescence double staining (400×). (*n* = 3). (**G**) Percentage of pancreatic islet alpha cells. (*n* = 3). (**H**) Hepatic glycogen content. (*n* = 10). (**I**) Hepatic insulin receptor substrate1 (IRS1), insulin receptor substrate2 (IRS2), and glucose transporter 2 (GLUT2) mRNA levels. (*n* = 3). (**J**) Representative graph of pancreatic IRS2 immunofluorescence. (*n* = 3). Compared with the normal group, ^▲^*P <* 0.05, ^▲▲^*P <* 0.01; compared with the model group, *P < 0.05, **P < 0.01. NG: normal control group; MG: model control group; BUDE-L: BUDE low-dose (75 mg·kg^− 1^) group; BUDE-H: BUDE high-dose (150 mg·kg^− 1^) group

We performed glucose tolerance tests to assess glucose homeostasis. The rats in each group displayed elevated blood glucose levels following a glucose load. The blood glucose concentrations of rats in each group peaked at 1 h. At 1 and 2 h, the blood glucose concentrations of rats in the model group exhibited noticeably more elevated than those of the norm (*P* < 0.01, 0.05). However, the BUDE-H significantly decreased blood glucose concentrations in model rats (*P* < 0.05). The AUC results indicated a significantly greater AUC in the model group compared to the normal group (*P* < 0.01). However, when BUDEs were administered, the AUC significantly declined in comparison towards the modeling group (*P* < 0.01, 0.05) (Fig. 4A, B).

Rats in the model group had considerably higher fasting insulin levels than rats in the normal group (*P* < 0.01). In contrast to the model group, the model rats’ fasting insulin levels dropped markedly after receiving BUDEs (*P* < 0.01, 0.05). Meanwhile both doses can significantly improve insulin resistance, and increased insulin sensitivity index (Fig. 4C-E). The islet β-cells in the model group were enlarged and deformed, and the proportion of islet α-cells was marked elevated in contrast to the normal group (*P* < 0.05). BUDE significantly improved the abnormal proliferation of islet β-cells and the de-differentiation of β-cells into α-cells in the model rats (*P* < 0.05) (Fig. 4F, G). These results suggest that BUDE can improve the function of pancreatic islet cells to maintain the body’s blood glucose homeostasis.

The hepatic glycogen content results indicated a significant reduction in the model group in contrast to the normal (*P* < 0.01). All BUDE groups, however, marked increased hepatic glycogen content (*P* < 0.01) (Fig. 4H). The liver mRNA analysis revealed significantly decreased levels of insulin receptor substrate1 (IRS1), insulin receptor substrate12 (IRS2), and glucose transporter 2 (GLUT2) in the model group as opposed to the normal group (*P* < 0.01). Nevertheless, all groups of BUDE showed a significant increase in the mRNA levels of liver IRS1, IRS2, and GLUT2 (*P* < 0.01, 0.05) (Fig. 4I). Furthermore, pancreatic immunofluorescence showed that BUDE markedly increased pancreatic IRS2 expression (Fig. 4J). The aforementioned results reveal that there might turn out improvements in the pancreatic and liver’s insulin resistance. Improved general metabolic health could result from this.

#### BUDE inhibit LPS-induced cellular inflammatory factor levels

**Fig. 5:**
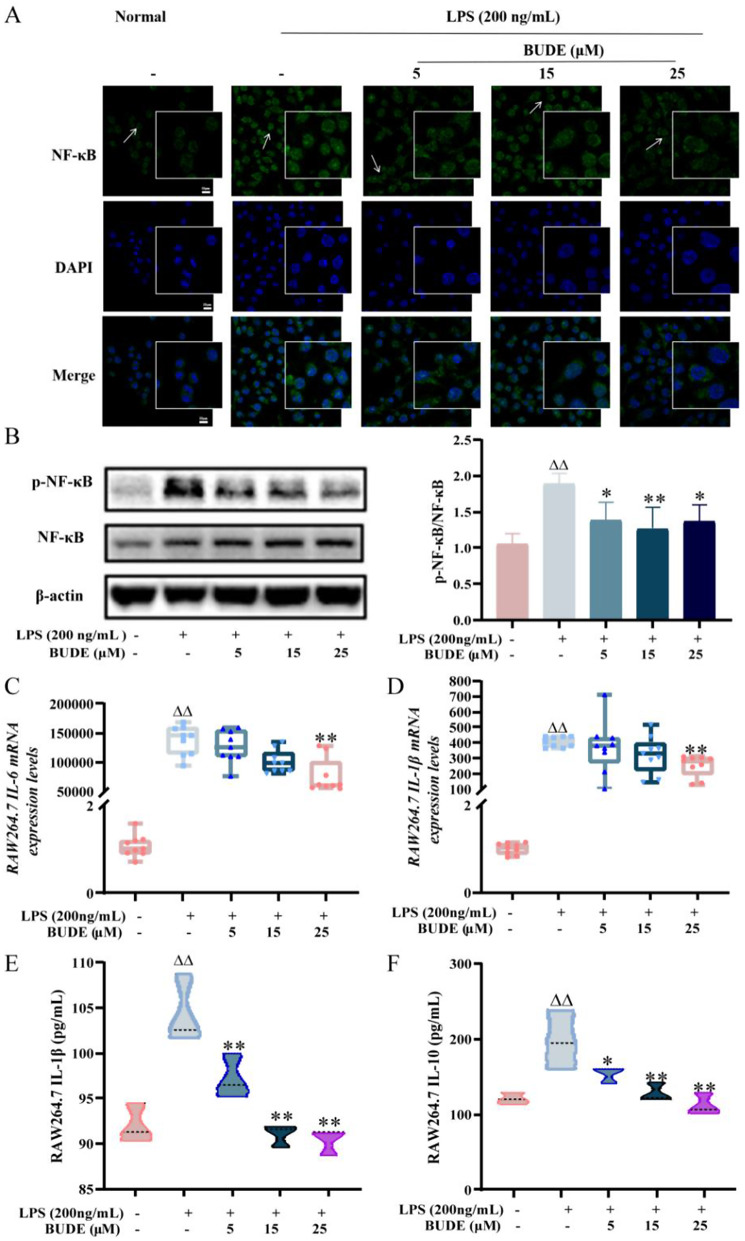
Effect of BUDE on inflammation in model cells. (**A**) Representative graph of NF-κB immunofluorescence of RAW264.7 cells (630×). (*n* = 3). (**B**) Representative maps of RAW264.7 cell NF-κB protein blots and relative expression levels of p-NF-κB protein. (*n* = 3). (**C**-**D**) Cellular IL-6, IL-1β mRNA expression levels. (*n* = 3). (**E**-**F**) Cellular supernatant IL-1β, IL-10 expression levels. (*n* = 3). Compared with the normal group, ^▲^*P <* 0.05, ^▲▲^*P <* 0.01; compared with the model group, *P < 0.05, **P < 0.01

In comparison to the normal group, RAW264.7 cells in the model group showed enlarged morphology, and there was a noticeable increase in the green fluorescence of NF-κB p65 within the nucleus. In contrast, treatment with BUDE prevented NF-κB p65 from moving to the nucleus, which suppressed the triggering of the NF-κB p65 signaling pathway (Fig. 5A). Additionally, the expression of p-NF-κB p65 protein was marked elevated in RAW264.7 cells of the model group compared to normal cells, but administration of BUDE significantly decreased the level of NF-κB p65 protein phosphorylation (*P* < 0.01, 0.05) (Fig. 5B). Furthermore, in RAW264.7 cells, the model group’s levels of IL-1β and IL-6 mRNA expression were far greater than those within the normal group (*P* < 0.01). A high dose of BUDE (25 µM) markedly decreased the levels of inflammatory factor mRNA expression in model rats (*P* < 0.01) (Fig. 5C, D). The model group had notably greater levels of IL-10 and IL-1β than normal RAW264.7 cells (*P* < 0.01). Conversely, in all BUDE dosage groups, the levels of IL-10 and IL-1β were considerably lower than in the model group (*P* < 0.01, 0.05) (Fig. 5E, F).

## Discussion

MetS is centralized by insulin resistance [27], and its causative factors are complex, mainly caused by poor lifestyle and dietary intolerance [28, 29]. Hence, we constructed a model of MetS using high-fat and high-sugar compounded excessive alcohol consumption, and administered the drug for 6 weeks while modeling to explore the mechanism of preventing MetS. We started from the main cause of metabolic abnormality to address the metabolic disorders caused by inflammation as well as insulin resistance. When compared with the model group, both high and low doses of BUDE prevented the elevation of blood pressure, reduced the expression of lipids, blood glucose, and inflammatory factors to varying degrees, and improved insulin resistance, in addition to pancreatic islet β-cells and liver hepatocyte damage.

The development of MetS is significantly influenced by an unhealthy diet. In metabolically disordered rats, nutrients and products of metabolic excess (LPS) migrate from the intestine into the bloodstream, which will then activate macrophage over-activation in tissues [30], contributing to an imbalance in the number of M1 and M2 macrophages, where the pro-inflammatory factors secreted by the M1-type macrophages will activate other inflammatory pathways to form inflammatory waterfalls [31, 32], resulting in malignant inflammatory infiltration. Macrophage secretion of inflammatory mediators can promote vasodilatation and increase vascular permeability, attracting other immune cells to the site of inflammation and creating a localized infiltrate of inflammation. At the same time, macrophages can also spread the local inflammatory response throughout the body by interacting with other immune cells, such as T cells and B cells, triggering a systemic inflammatory response. Major metabolic tissues (liver, etc.), while being damaged by inflammatory factors, also secrete exosomes to communicate with peripheral cells as well as distant organs, triggering more macrophage aggregation and polarization [33, 34], affecting systemic metabolism, and ultimately triggering systemic low and slow-grade inflammation.

Our study found that the anti-inflammatory effect of BUDE can hit the ideal target for breaking the vicious cycle, with properties that effectively alleviate the root causes of inflammation. Drawing on BUDE’s capability to reduce the penetration of LPS into the bloodstream by enhancing the intestinal barrier in our previous study, our current phase of research has revealed that BUDE can inhibit the phosphorylation of NF-κB in M1-type macrophages, suppress the expression of IL-1β and IL-6. This action reduces various tissues damage caused by inflammatory factors, decreases LPS, hs-CRP and IL-6 levels in rat blood, and prevents the amplification of inflammatory responses. Ultimately, it regulates the state of systemic chronic low-grade inflammation. It is important to highlight that while a positive correlation between BUDE efficacy and dosage was observed in our in vitro experiments, this relationship was not evident in our in vivo experiments. This disparity may be attributed to the variations between in vivo and in vitro environments.

The anti-inflammatory effect of BUDE is likely predominantly associated with buddleoside. Additionally, our study using UPLC-Q-TOF-MSE revealed other components, among which acacetin [35, 36], apigenin [37], luteolin [38, 39], and chlorogenic acid [40] have all been reported to have anti-inflammatory effects, and all of these components may favor the anti-inflammatory effects of BUDE. It has been shown that phenolic fractions of peppermint (containing buddleoside) [41] as well as chlorogenic acid [42] inhibit lipopolysaccharide (LPS)-induced pro-inflammatory production and the effect of NF-κB activation in RAW264.7 cells to explore the mechanism of inflammatory activity which is consistent with our results.

Although inflammation is most directly responsible for inducing vascular endothelial dysfunction and causing elevated blood pressure, it is also an important factor in inducing insulin resistance. It is important to note that the relationship between insulin resistance and inflammation is reciprocal [43], i.e., inflammation can cause insulin resistance, and at the same time, insulin resistance may trigger or exacerbate inflammation. Insulin resistance is present throughout the MetS, and hyperinsulinemia triggered by compensatory increase in insulin is another important cause of induced hypertension [44]. Excessive consumption of fat, sweet and thick flavors will lead to the body to produce a large number of inflammatory factors, inflammatory cytokines trigger a cascade of inflammatory mediators, further damage to the liver, pancreas and other tissues, and deteriorate their insulin sensitivity, triggering insulin signaling disorders, leading to insulin resistance and causing metabolic abnormalities.

Excessive consumption of sugar, fat, and alcohol can disrupt the balance of intestinal flora, leading to elevated production of LPS, a byproduct of intestinal metabolism. This disruption can also increase intestinal permeability, allowing LPS to enter the bloodstream more easily [45]. Our previous research has shown that BUDE can help restore healthy intestinal flora and strengthen the intestinal barrier, thereby lowering blood LPS levels [23]. However, elevated blood LPS levels are also closely linked to liver function. When the liver is compromised, it may struggle to effectively process the LPS released from the intestine, leading to a buildup of circulating LPS levels. Elevated levels of circulating endotoxin have been shown to correspond with the severity of liver disease in animal studies [46]. The liver is crucial in regulating metabolism and has the highest proportion of macrophages among all organs, with resident tissue macrophages playing a vital role in coordinating the inflammatory response in this tissue [47]. Hyperpolarization of macrophages results in excessive production of inflammatory factors, disrupts the expression of crucial proteins in the insulin signaling pathway, and significantly contributes to the development of hepatic insulin resistance [48]. Thus, blocking these intracellular inflammatory pathways prevents hepatic insulin resistance [49] and increases the likelihood of macrophage-mediated tissue-autonomous insulin resistance. In insulin resistance, abnormal hepatic insulin action is thought to be the main driver of insulin resistance, requiring higher circulating insulin levels to adequately control blood glucose levels [50]. In response to liver damage, hepatokines are released and have gained increased attention as regulators of metabolic diseases [33]. They can impact glucose and lipid metabolism in the body [51, 52], and are closely associated with insulin resistance [53] and inflammatory responses [54], thereby governing systemic metabolic balance and exerting broad effects on the entire organism [55]. It has been shown that apigenin [56] and baicalein, a dietary flavonoid found in various edible plants [57], can modulate hepatokines secretion and alleviate insulin resistance. This implies that BUDE may potentially improve metabolic disorders by influencing hepatokines secretion. In our study, BUDE was found to improve hepatic injury such as hepatic lipid deposition and fat vacuolization, decrease the expression of inflammatory factor IL-6, as well as increase the mRNA levels of hepatic insulin IRS1, IRS2, and GLUT2, in order to improve the metabolic insulin resistance in metabolically hypertensive rats, and to increase the accumulation of hepatic glycogen content followed by a significant decrease in the TC, LDL-c, and FBG levels of the model rats in order to improve glucose-lipid metabolism disorders.

The chronic inflammatory state occurs in the liver and it’s also occurs in the pancreatic tissue. When a large macrophage infiltration is present in the pancreatic tissue, it results in the production of inflammatory mediators such IL-1β and IL-6, which further exacerbates pancreatic tissue damage. Although the existence of pancreatic islets inherent immune cells in a certain degree of nutrients and metabolic excess in the production of a small amount of IL-1β can promote the function and survival of pancreatic islets [58], but the β-cells are in a prolonged period of excessive stress, this regulatory inflammation will be developed in the opposite direction, excessive inflammatory factors will damage the β-cells, interfere with insulin signaling pathways, leading to decreased insulin sensitivity of pancreatic β-cells and affecting pancreatic β-cell function. Worst of all, the triggered glucose homeostasis will be disrupted, and slightly elevated blood glucose levels will affect the ability of β-cells to divide and grow, as well as their vulnerability to inflammation [59], leading to dedifferentiation and a vicious cycle of pancreatic islet dysfunction. In our study, BUDE reduced the inflammatory infiltration of inflammatory cells in pancreatic islet cells, held back the NF-κB pathway from being activated, decreased the phosphorylation level of NF-κB, and subsequently reduced the overexpression of IL-1β and IL-6 in the pancreas, thus preventing the over-activation of pancreatic β-cells that leads to aberrant division and growth, improving the dysfunction of pancreatic β-cells, and decreasing over-compensation of insulin secretion. At the same time, it can also inhibit the reduction and catabolism of IRS2 in pancreatic β-cells, improve insulin resistance in the pancreas, and ultimately improve the disruption of glycolipid metabolism in model rats.

## Conclusions

**Fig. 6:**
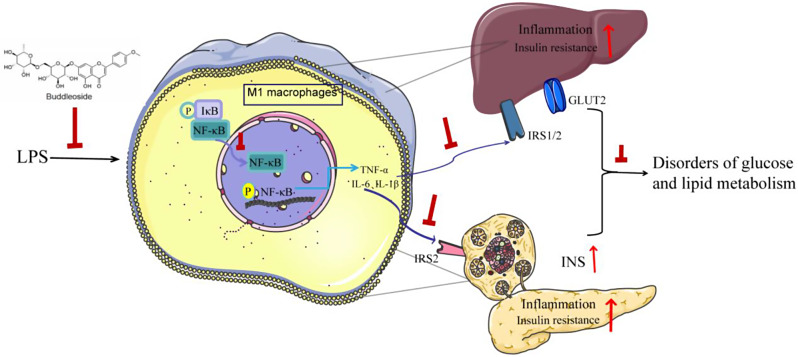
The graphic illustration of the mechanism of BUDEs ameliorating MetS

BUDE has been shown to have several beneficial effects on the body. They can reduce the presence of LPS, which is a type of endotoxin that can cause inflammation. Additionally, they can balance the polarization imbalance between pro-inflammatory and anti-inflammatory phenotypes of macrophages in the liver and pancreas. This aids in preventing the NF-κB pathway from being activated and lowers the expression of inflammatory factors. As a result, the liver and pancreas are protected from damage caused by inflammatory factors. This leads to improvements in pancreatic and hepatic insulin resistance, as well as better glucose and lipid metabolism. Furthermore, this can help to prevent the elevation of blood pressure and ultimately correct metabolic disorders. This ultimately serves to prevent MetS (Fig. 6).

### Electronic supplementary material

Below is the link to the electronic supplementary material.

Supplementary Material 1

## Data Availability

The datasets utilized in this manuscript are not accessible to the public. Requests to access the datasets should be directed to zjtcmlgy@163.com.

## Abbreviations

AUC: Area under curve
BSA: Bovine serum albumin
BUDE: Buddleoside-rich *Chrysanthemum indicum* L. extract
DBP: Diastolic blood pressure
FBG: Fasting blood glucose
FINS: Fasting insulin
GLUT2: Glucose transporter 2
H&E: Hematoxylin-eosin staining
HDL-c: High-density lipoprotein cholesterol
hs-CRP: High-sensitivity C-reactive protein
IF: Immunofluorescence
IHC: Immunohistochemistry
IL: Interleukin
IR: Insulin resistance
IRS: Insulin receptor substrate
ISI: Insulin sensitivity
LDL-c: Low-density lipoprotein cholesterol
LPS: Lipopolysaccharide
MBP: Mean arterial blood pressure
MetS: Metabolic syndrome
qRT-PCR: Quantitative real-time PCR
RAW264.7: Mouse mononuclear macrophages cells
SBP: Systolic blood pressure
TC: Total cholesterol
TG: Triglycerid
