# Supplementary material for: Buddleoside-rich Chrysanthemum indicum L. extract modulates macrophage-mediated inflammation to prevent metabolic syndrome induced by unhealthy diet
Source: BMC Complement Med Ther. 2024 Aug 23;24:315. doi: 10.1186/s12906-024-04583-2 (PMC11344343; doi:10.1186/s12906-024-04583-2)
Supplement: Supplementary file 1 — Supplementary Material 1 [file 12906_2024_4583_MOESM1_ESM.pdf]

Supplementary uncut and non-processed blots

Supplementary Original Western blots used for Fig.2F

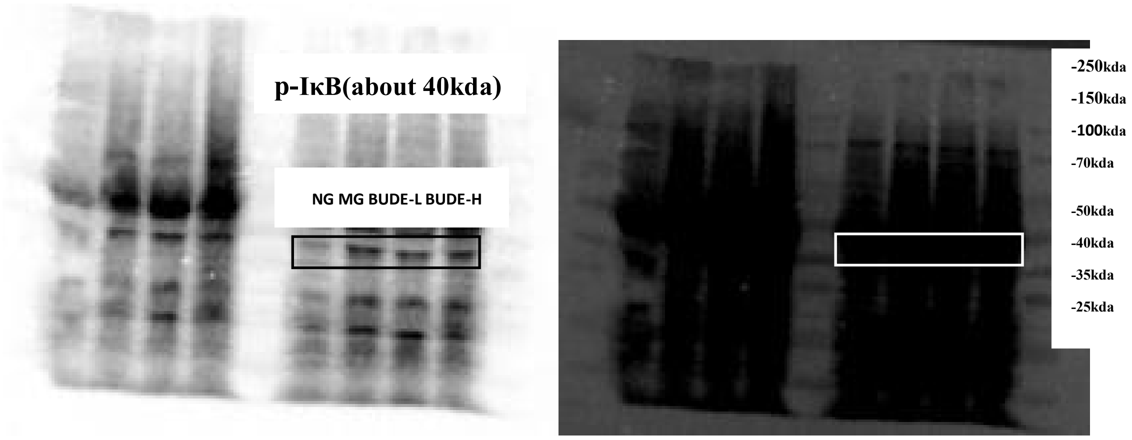

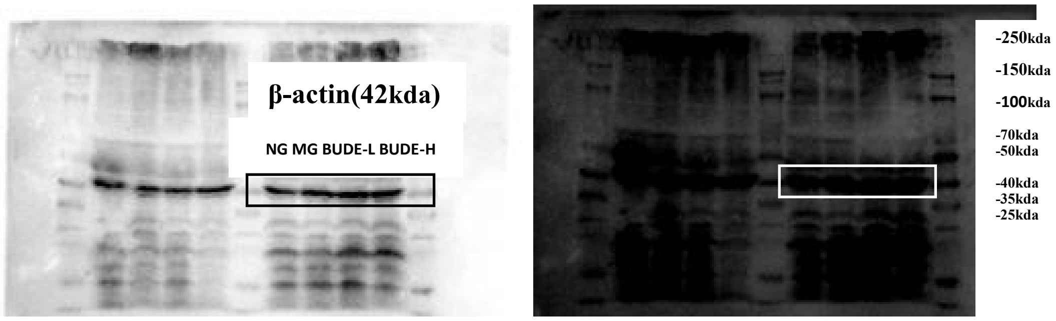

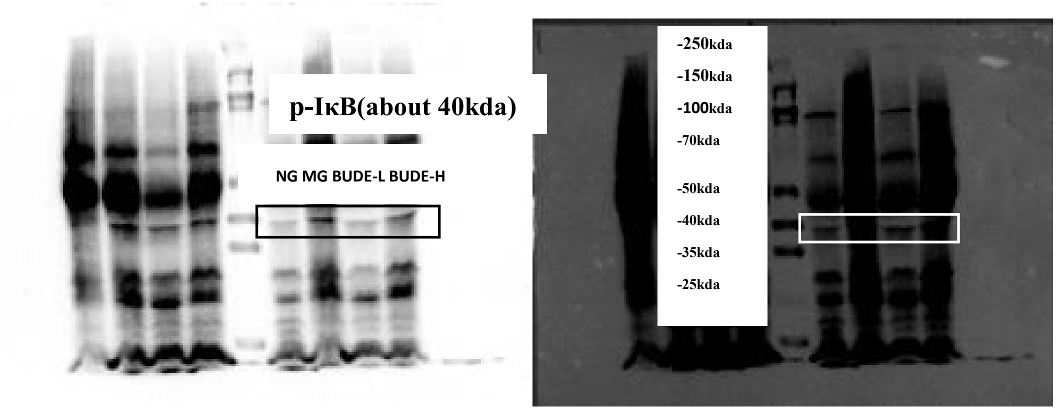
p-IκB(about 40kda)
NG MG BUDE-L BUDE-H
-250kda
-150kda
-100kda
-70kda
-50kda
-40kda
-35kda
-25kda

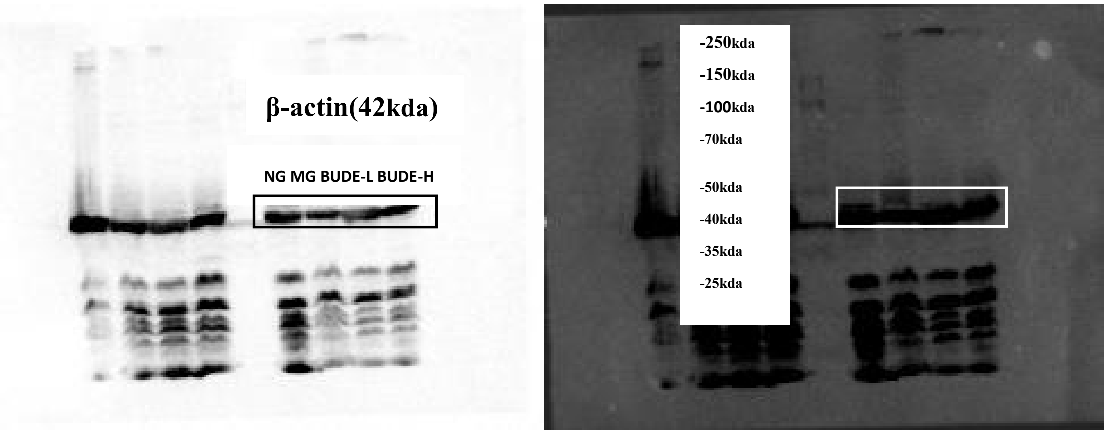
β-actin(42kda)
NG MG BUDE-L BUDE-H
-250kda
-150kda
-100kda
-70kda
-50kda
-40kda
-35kda
-25kda

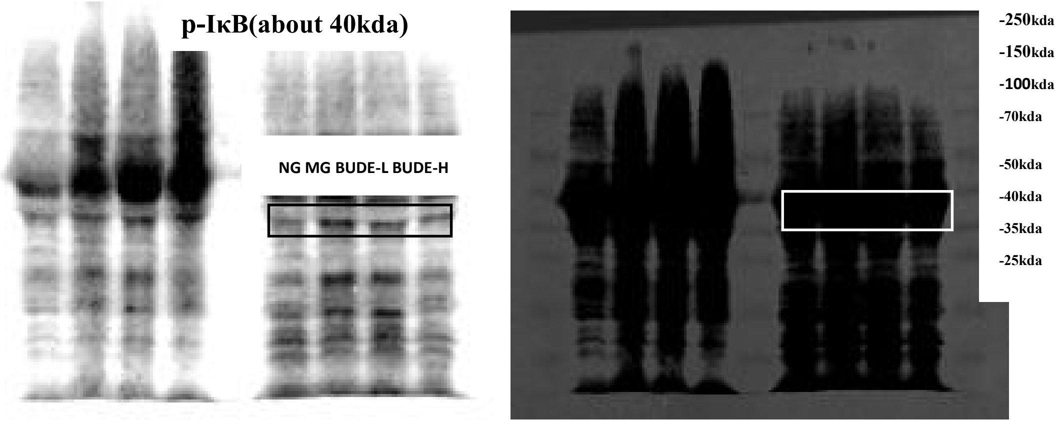
p-IκB(about 40kda)
NG MG BUDE-L BUDE-H
-250kda
-150kda
-100kda
-70kda
-50kda
-40kda
-35kda
-25kda

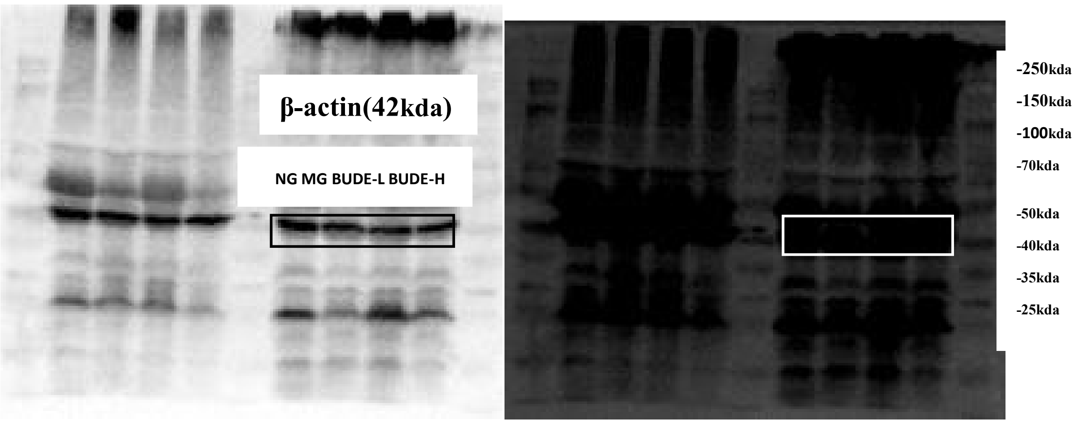

Fig.1 The uncut and and non-processed blot image of p-IKB and corresponding β-actin

Samples are labelled throughout as follows.

NG: normal group

MG: model group

BUDE-L: **BUDE** low dose group,75 mg·kg^{-1}

BUDE-H: **BUDE** high dose group,150 mg·kg^{-1}

This is the result of WB experiment with 3 samples in each group. All proteins are the same batch of samples. All bands were incubated with the target protein first followed by eluted with stripping buffer and finally incubated with β-actin.

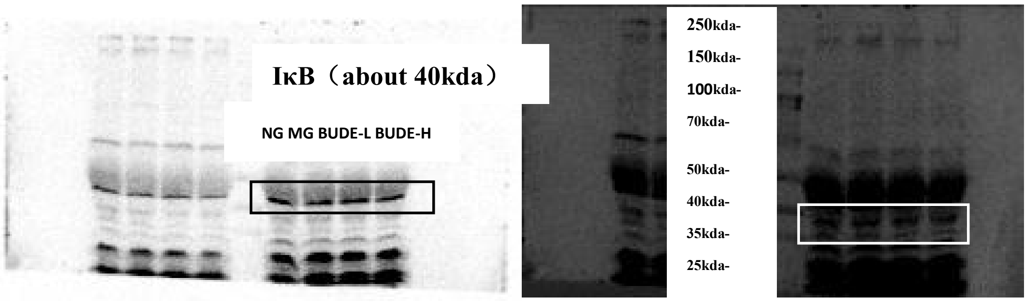

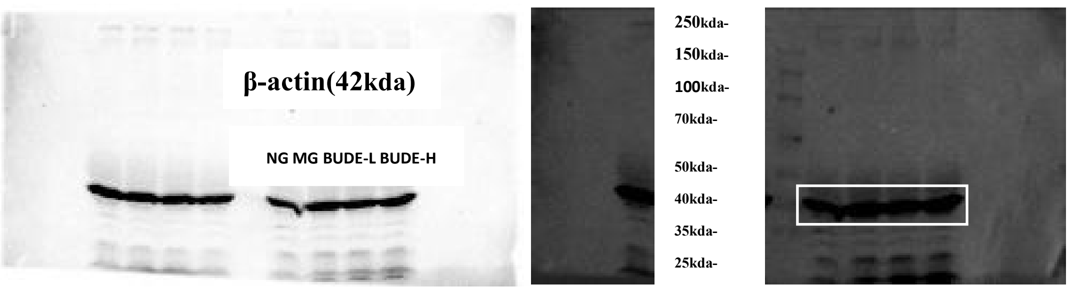

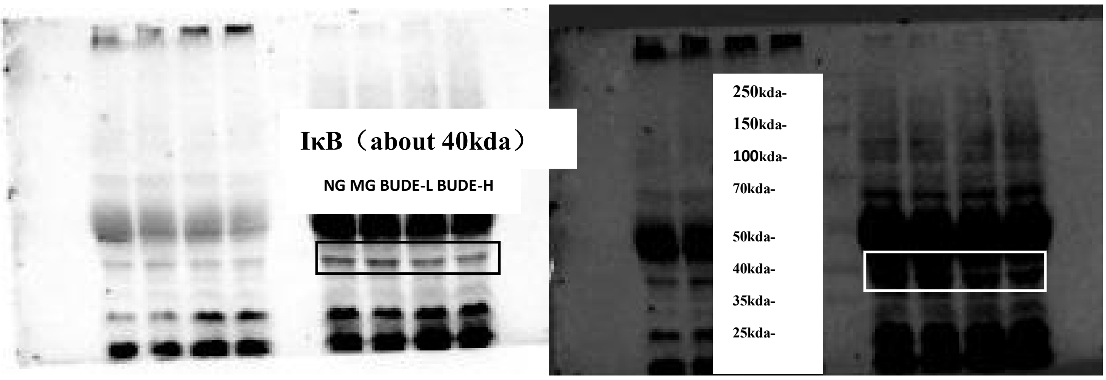
IκB（about 40kda）
NG MG BUDE-L BUDE-H
250kda-
150kda-
100kda-
70kda-
50kda-
40kda-
35kda-
25kda-

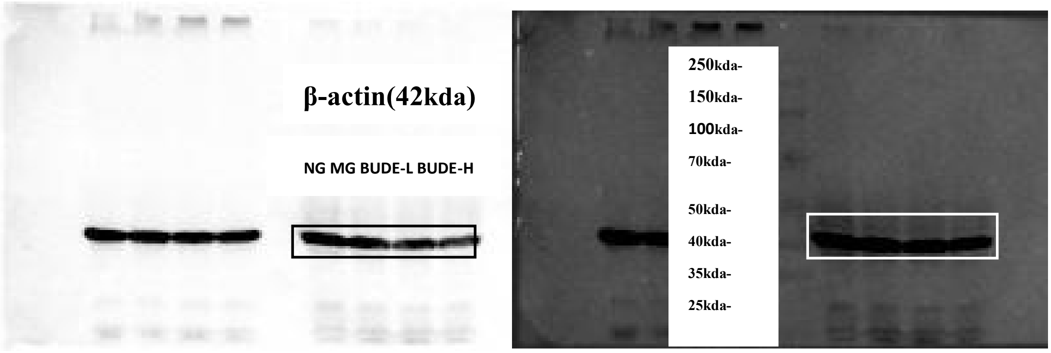
β-actin(42kda)
NG MG BUDE-L BUDE-H
250kda-
150kda-
100kda-
70kda-
50kda-
40kda-
35kda-
25kda-

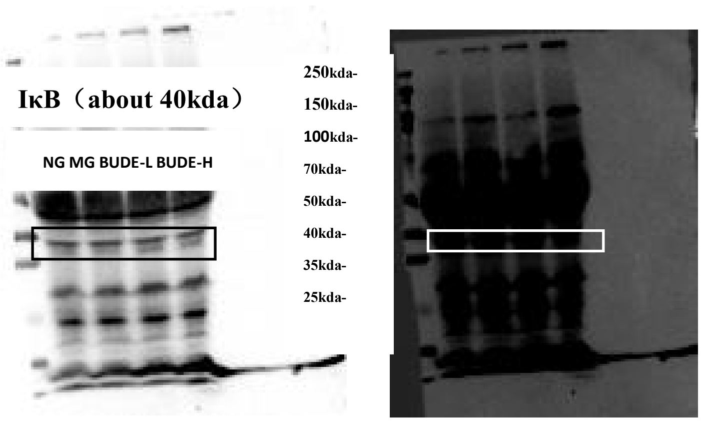
IκB（about 40kda）
NG MG BUDE-L BUDE-H
250kda-
150kda-
100kda-
70kda-
50kda-
40kda-
35kda-
25kda-

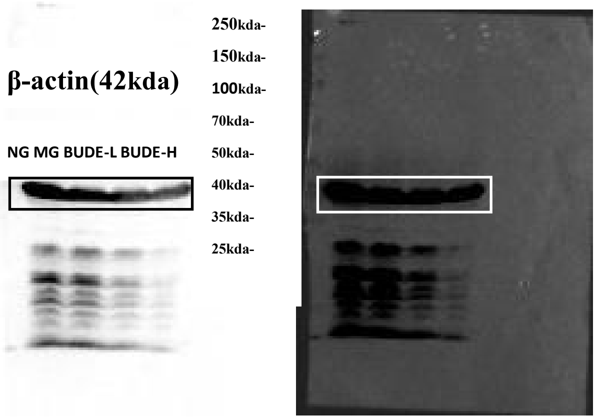

Fig.2 The uncut and and non-processed blot image of IκB and corresponding β-actin
Samples are labelled throughout as follows.

NG: normal group

MG: model group

BUDE-L: **BUDE** low dose group,75 mg·kg^{-1}

BUDE-H: **BUDE** high dose group,150 mg·kg^{-1}

This is the result of WB experiment with 3 samples in each group. All proteins are the same batch of samples.All bands were incubated with the target protein first followed by eluted with stripping buffer and finally incubated with β-actin.

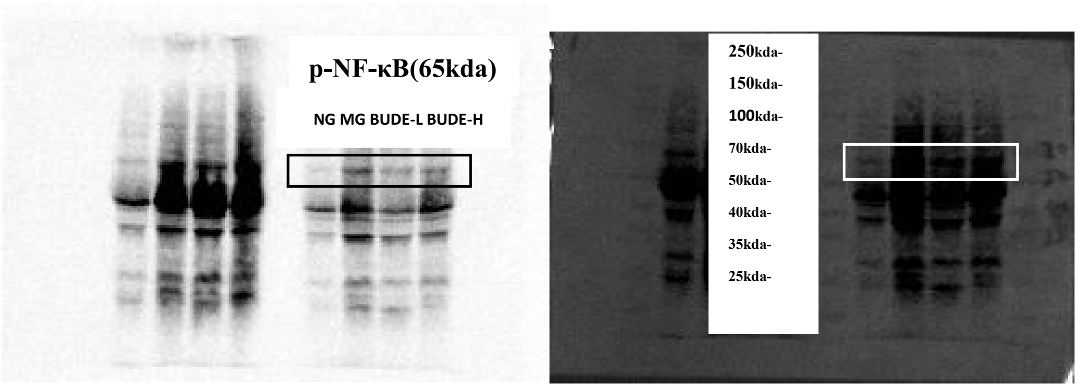

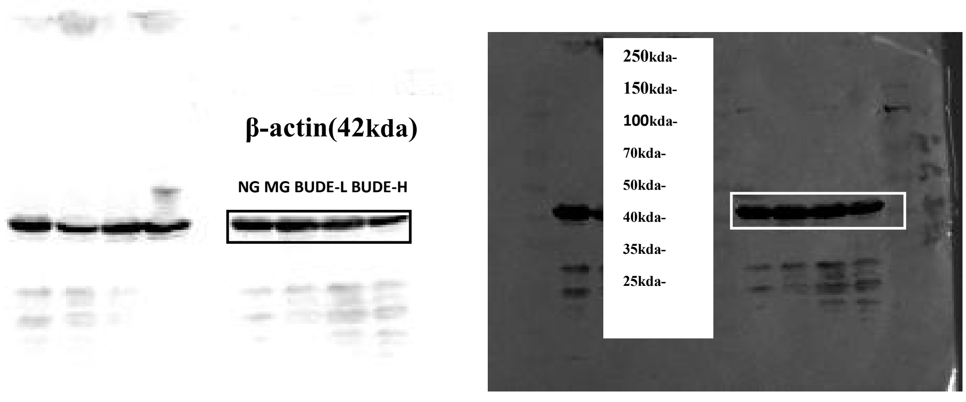

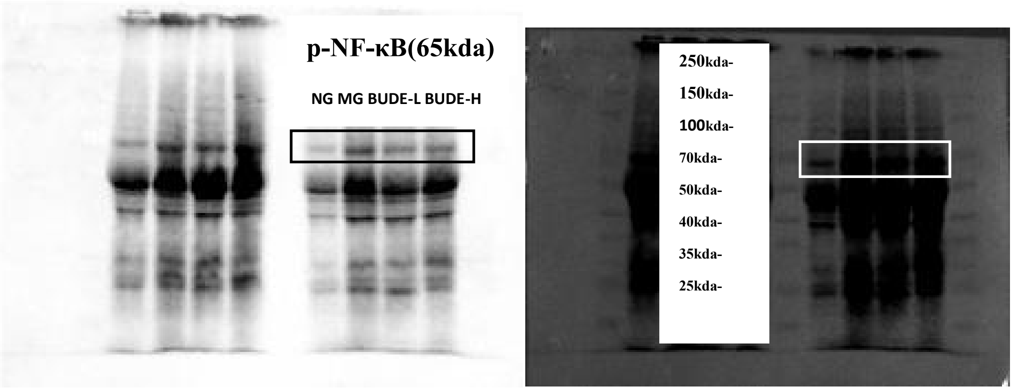
p-NF-κB(65kda)
NG MG BUDE-L BUDE-H
250kda-
150kda-
100kda-
70kda-
50kda-
40kda-
35kda-
25kda-

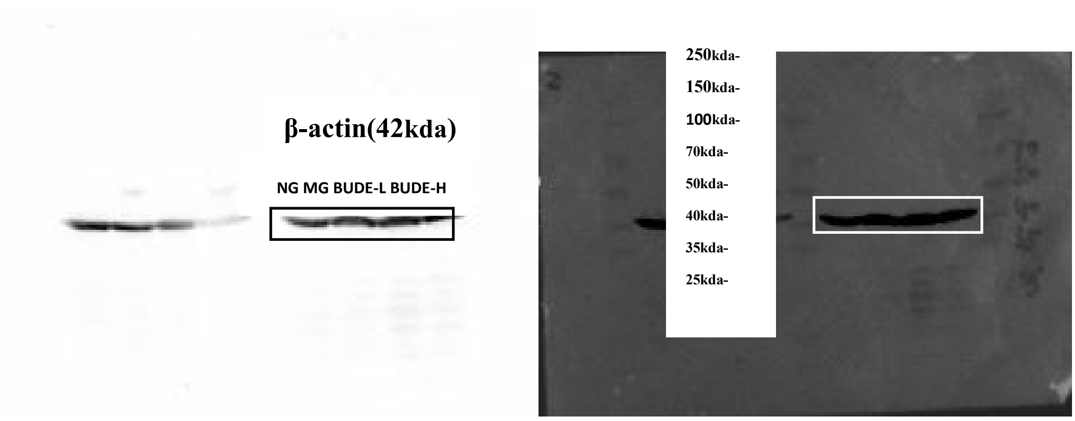
β-actin(42kda)
NG MG BUDE-L BUDE-H
250kda-
150kda-
100kda-
70kda-
50kda-
40kda-
35kda-
25kda-

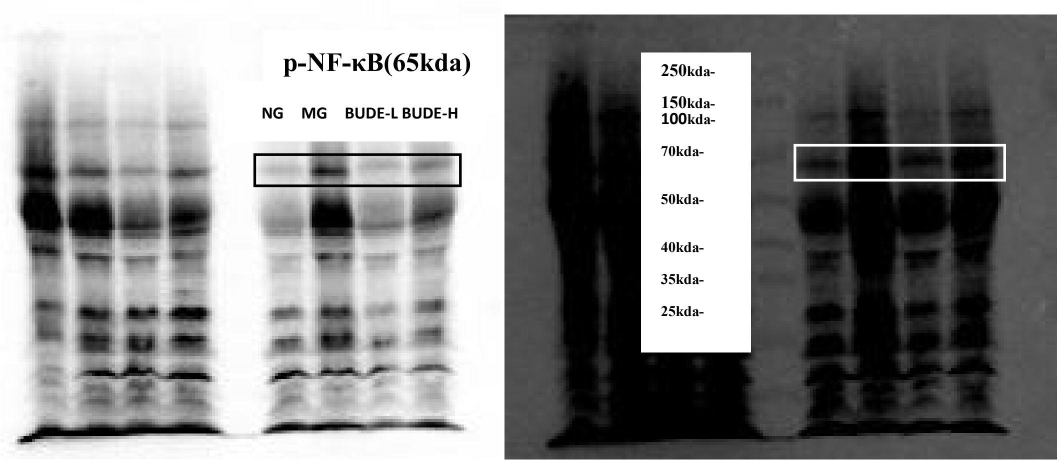
p-NF-κB(65kda)
NG MG BUDE-L BUDE-H
250kda-
150kda-
100kda-
70kda-
50kda-
40kda-
35kda-
25kda-

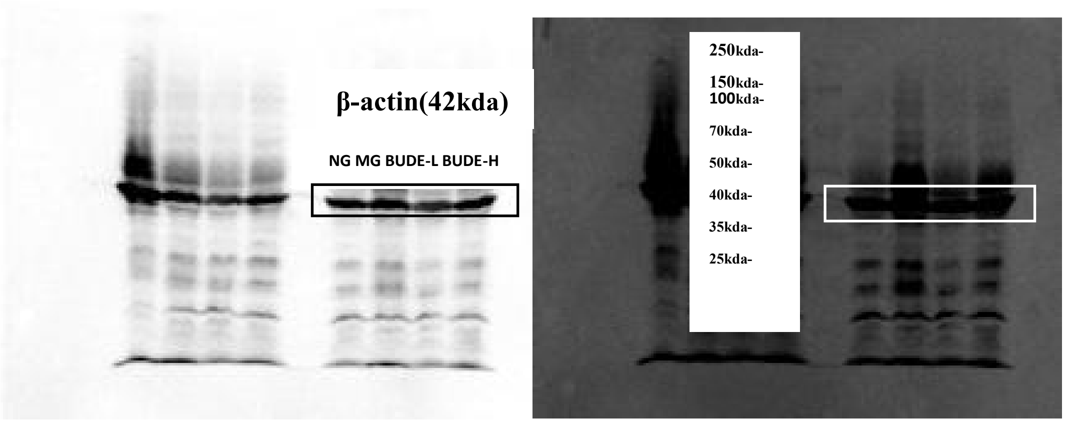

Fig.3 The uncut and and non-processed blot image of p-NF-κB and corresponding β-actin
Samples are labelled throughout as follows.

NG: normal group

MG: model group

BUDE-L: **BUDE** low dose group,75 mg·kg^{-1}

BUDE-H: **BUDE** high dose group,150 mg·kg^{-1}

This is the result of WB experiment with 3 samples in each group. All proteins are the same batch of samples.All bands were incubated with the target protein first followed by eluted with stripping buffer and finally incubated with β-actin.

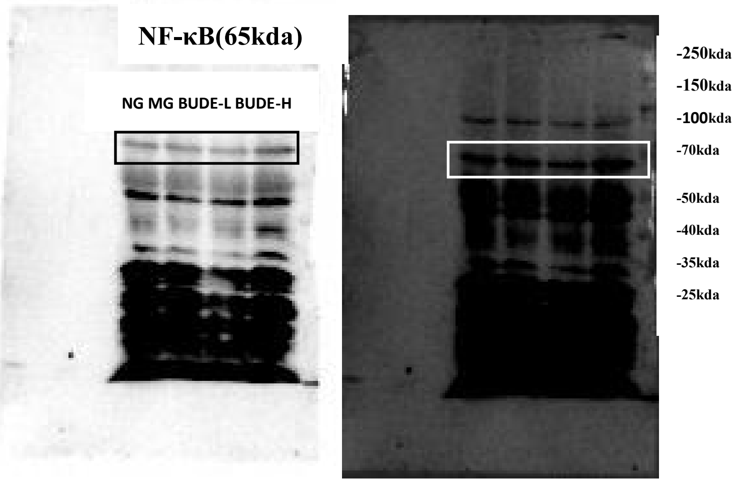

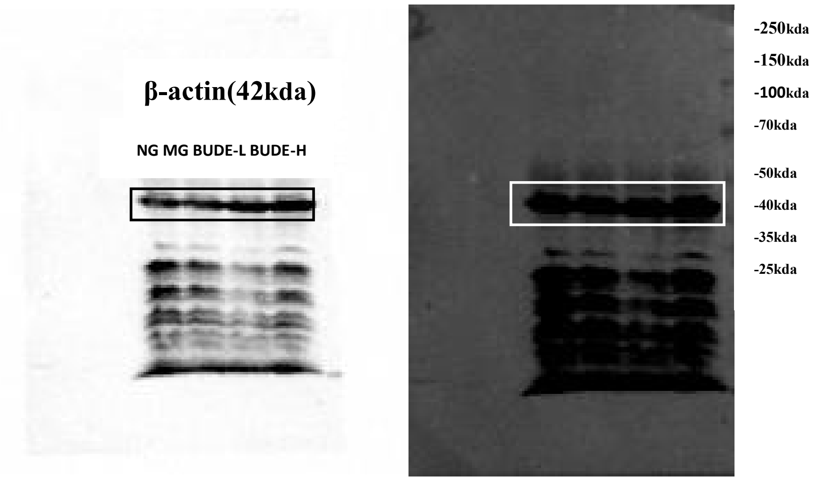
β-actin(42kda)
NG MG BUDE-L BUDE-H
-250kda
-150kda
-100kda
-70kda
-50kda
-40kda
-35kda
-25kda

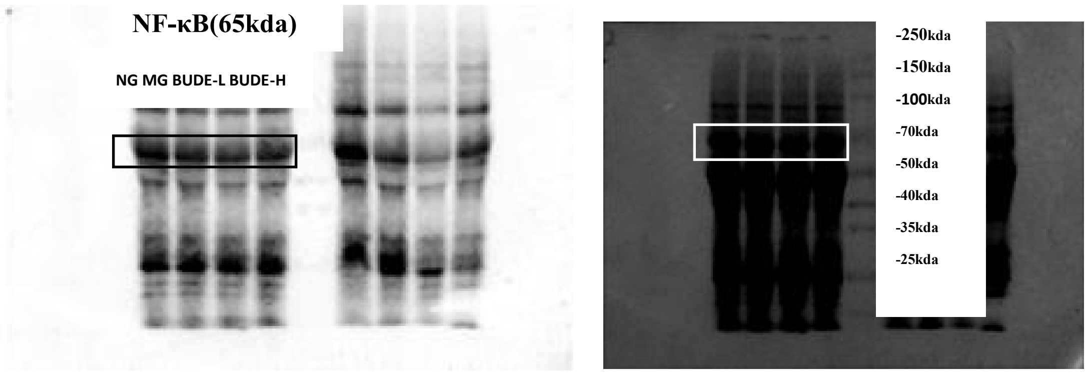
NF-κB(65kda)
NG MG BUDE-L BUDE-H
-250kda
-150kda
-100kda
-70kda
-50kda
-40kda
-35kda
-25kda

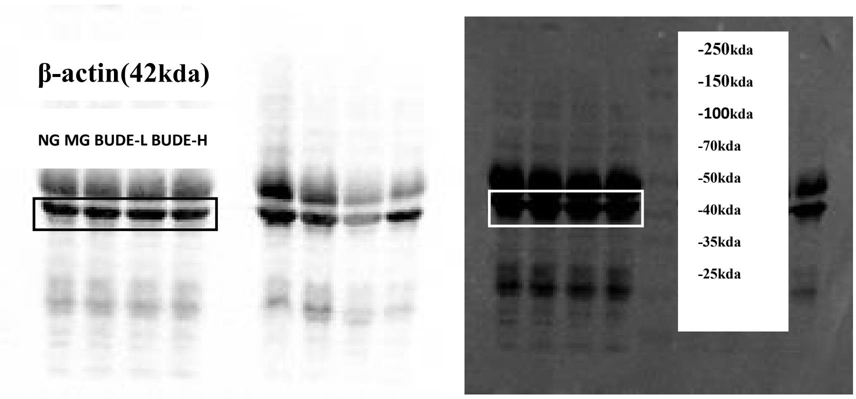
β-actin(42kda)
NG MG BUDE-L BUDE-H
-250kda
-150kda
-100kda
-70kda
-50kda
-40kda
-35kda
-25kda

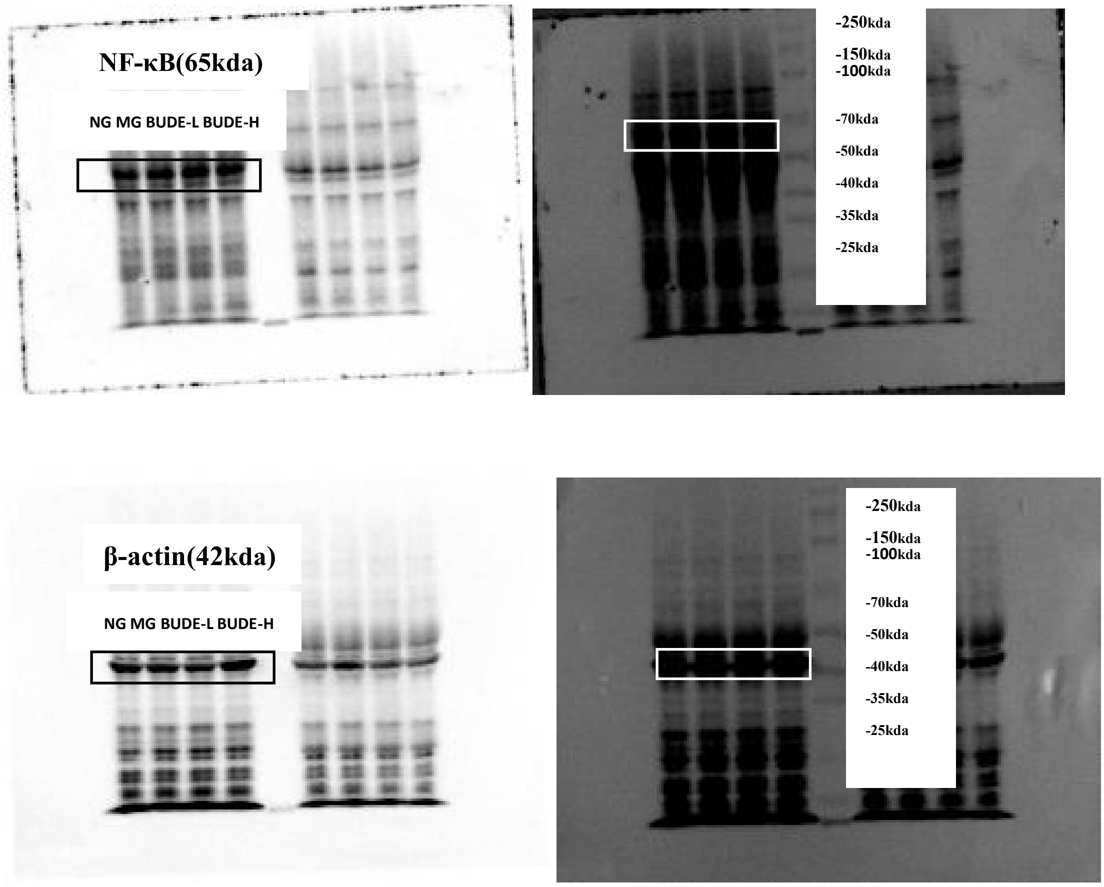

Fig.4 The uncut and and non-processed blot image of NF-κB and corresponding β-actin
Samples are labelled throughout as follows.

NG: normal group

MG: model group

BUDE-L: **BUDE** low dose group,75 mg·kg^{-1}

BUDE-H: **BUDE** high dose group,150 mg·kg^{-1}

This is the result of WB experiment with 3 samples in each group. All proteins are the same batch of samples. All bands were incubated with the target protein first followed by eluted with stripping buffer and finally incubated with β-actin.

Supplementary Original Western blots used for Fig.4B

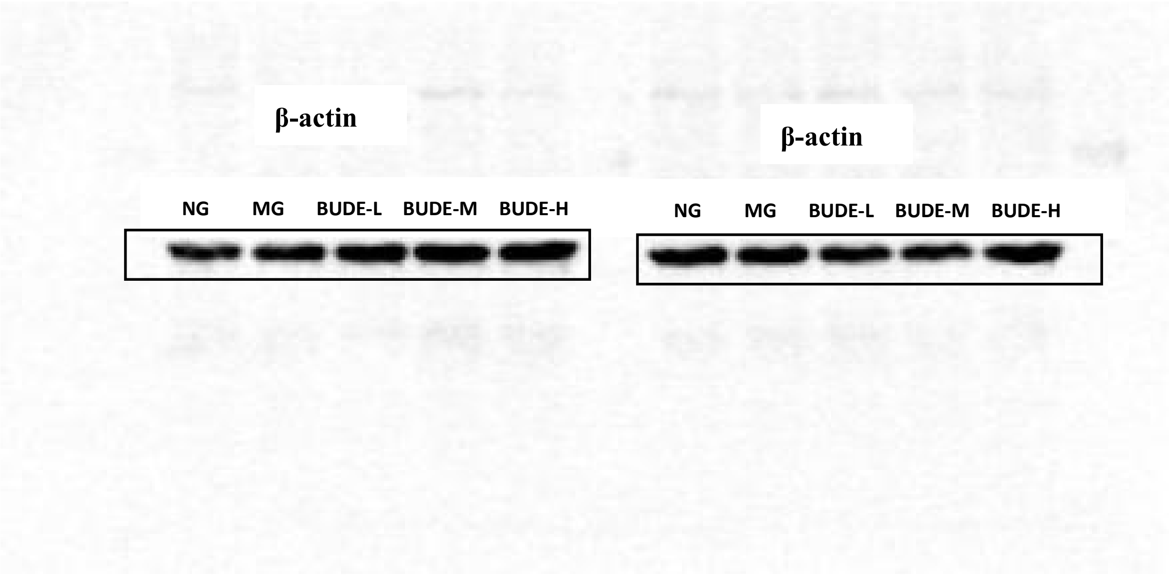
β-actin
β-actin
NG MG BUDE-L BUDE-M BUDE-H
NG MG BUDE-L BUDE-M BUDE-H

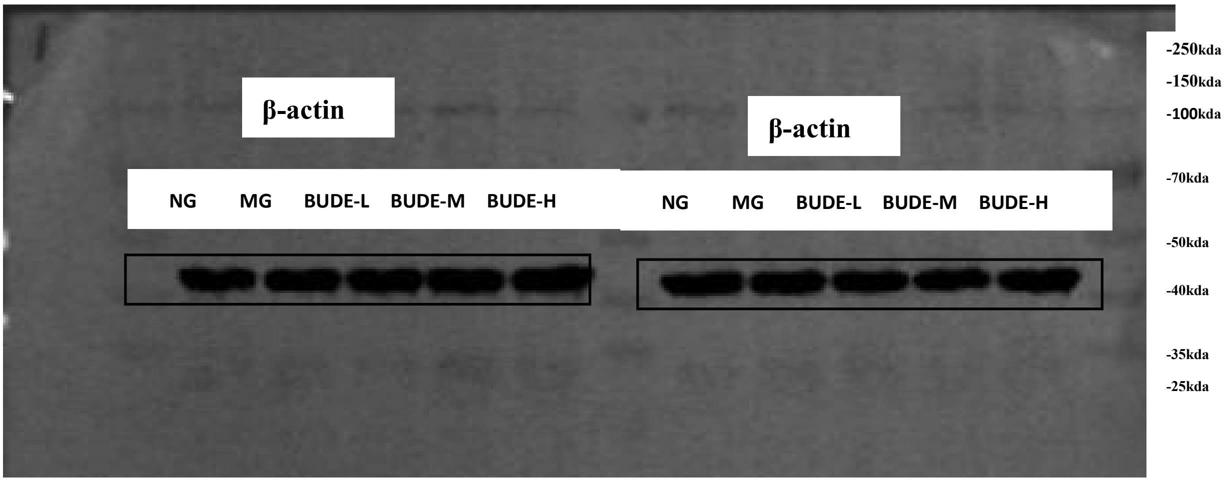
β-actin
β-actin
NG MG BUDE-L BUDE-M BUDE-H
NG MG BUDE-L BUDE-M BUDE-H
-250kda
-150kda
-100kda
-70kda
-50kda
-40kda
-35kda
-25kda

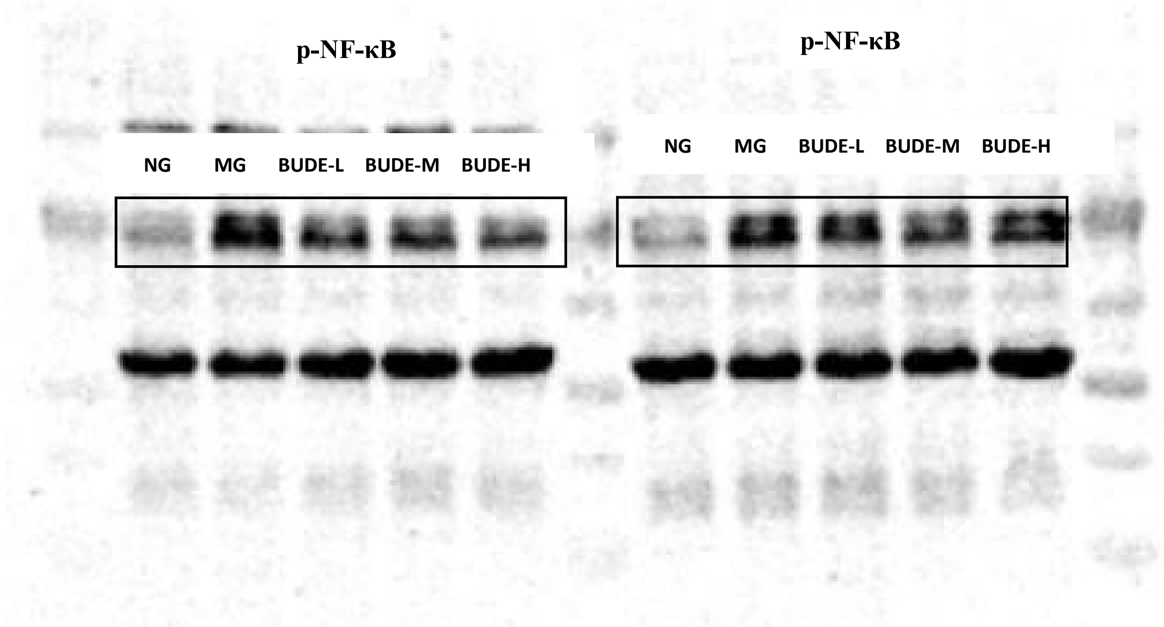
p-NF-κB
p-NF-κB
NG
MG
BUDE-L
BUDE-M
BUDE-H
NG
MG
BUDE-L
BUDE-M
BUDE-H

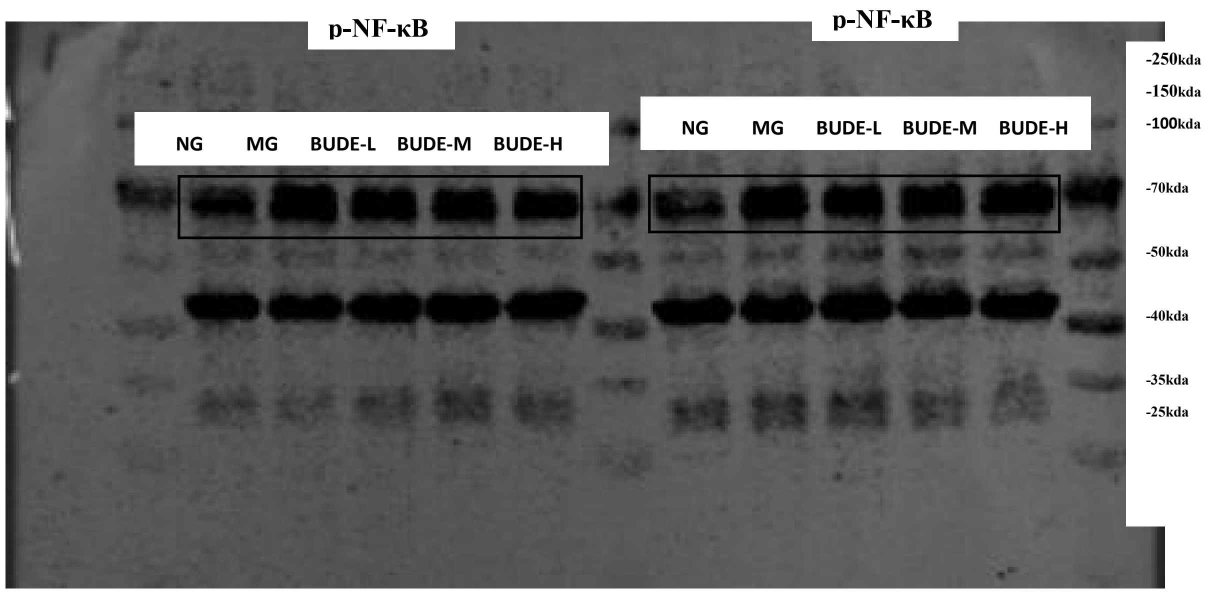
p-NF-κB
p-NF-κB
NG
MG
BUDE-L
BUDE-M
BUDE-H
NG
MG
BUDE-L
BUDE-M
BUDE-H
-250kda
-150kda
-100kda
-70kda
-50kda
-40kda
-35kda
-25kda

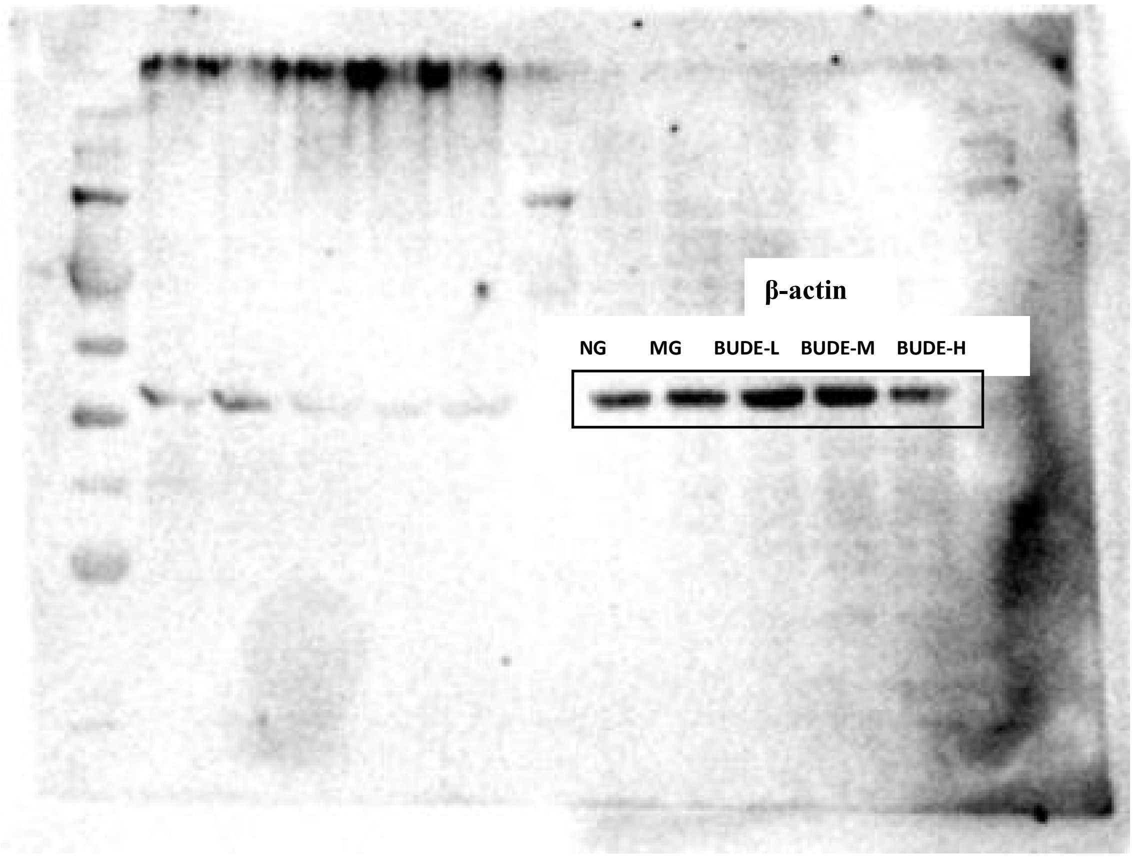
β-actin
NG
MG
BUDE-L
BUDE-M
BUDE-H

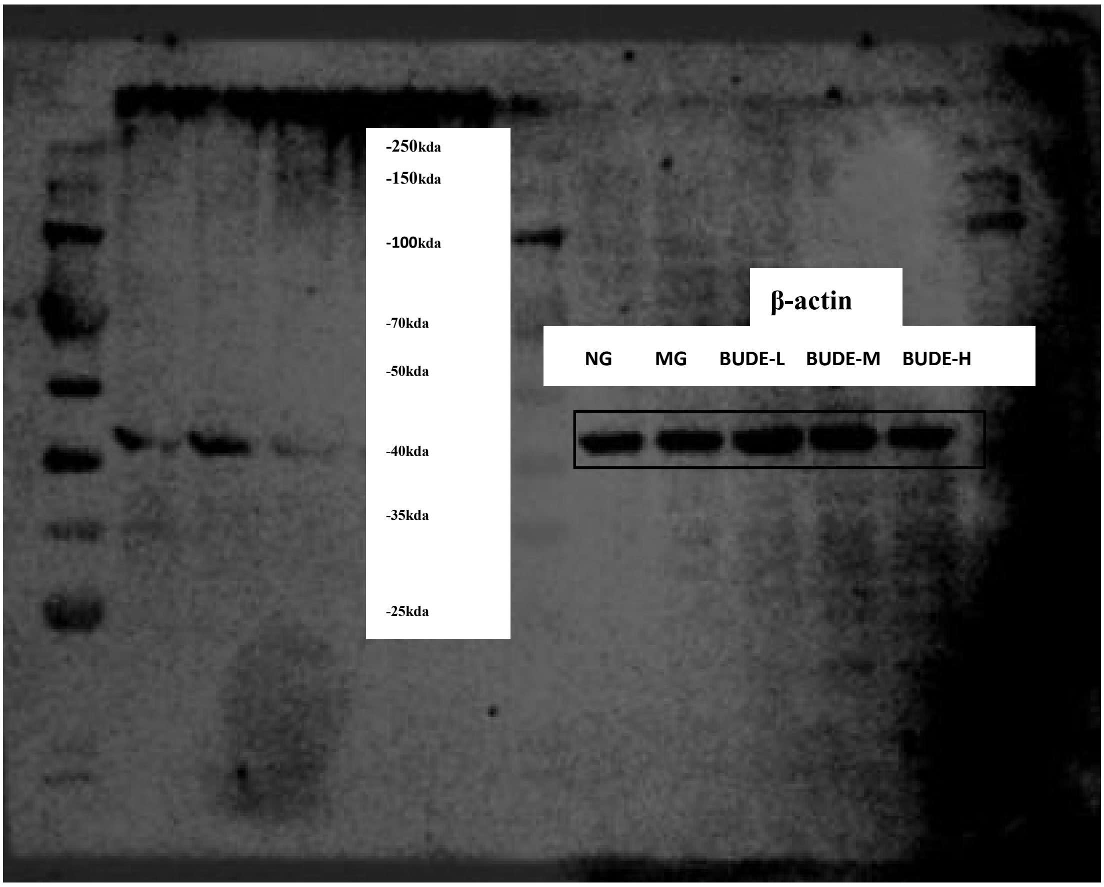
-250kda
-150kda
-100kda
-70kda
-50kda
-40kda
-35kda
-25kda
β-actin
NG
MG
BUDE-L
BUDE-M
BUDE-H

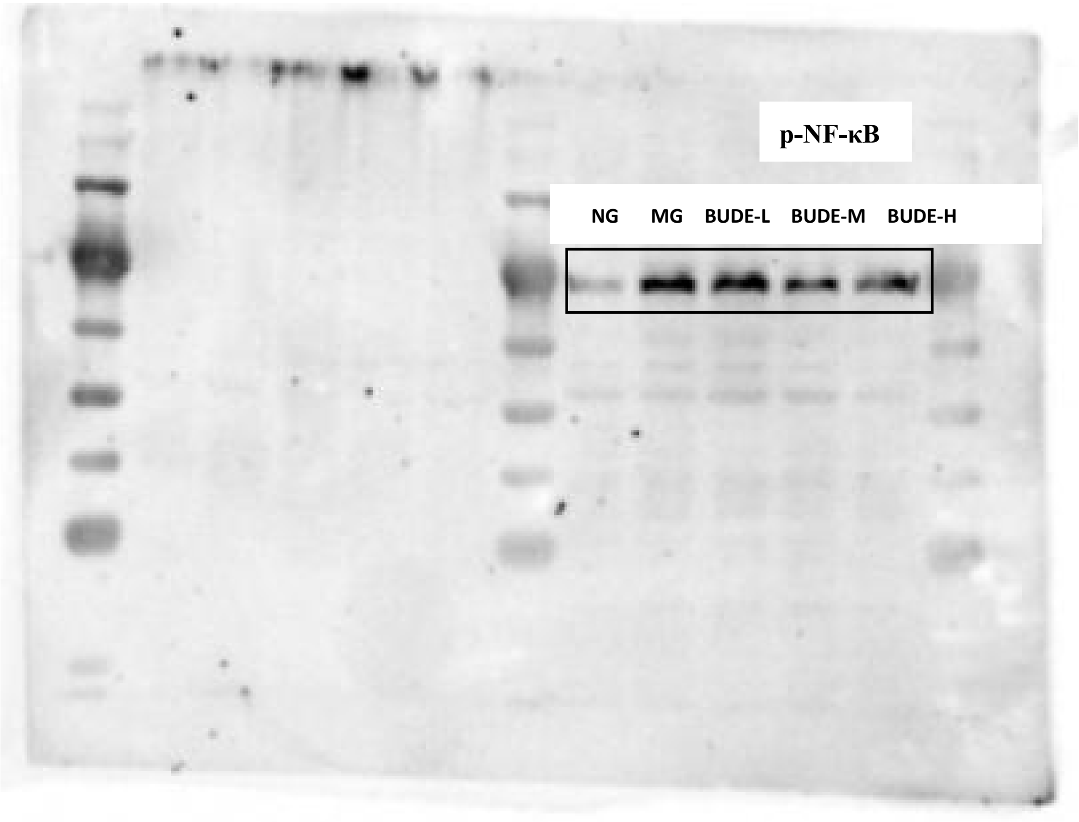

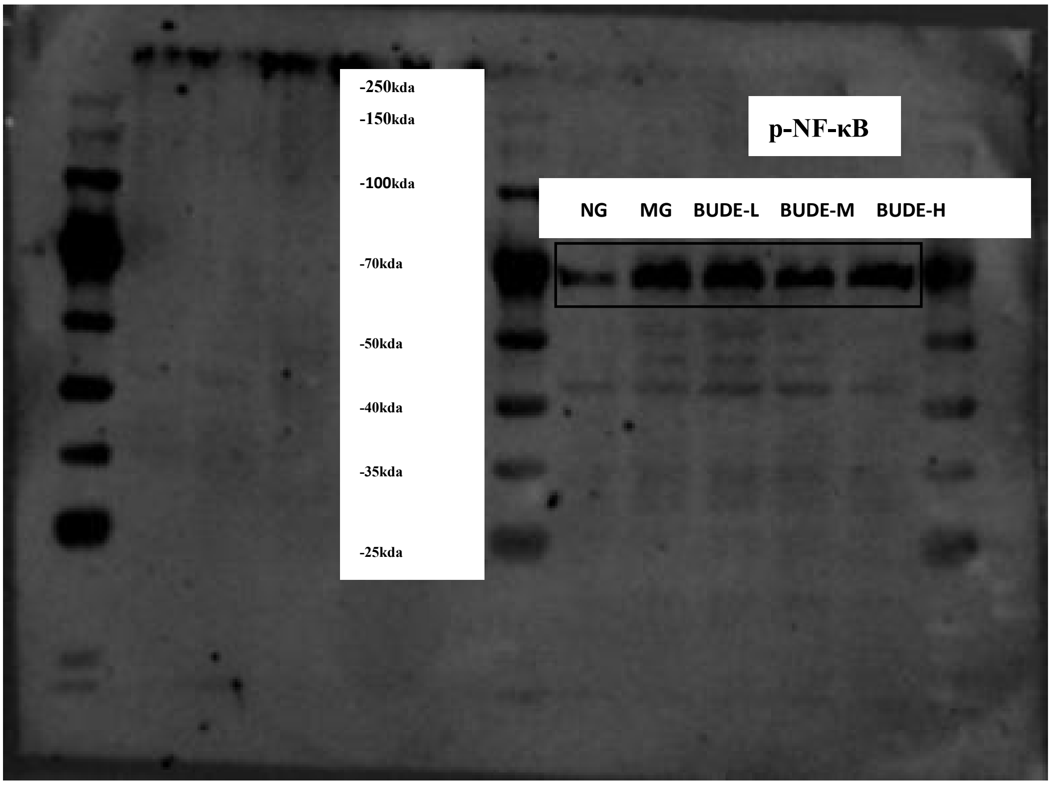

Fig.1 The uncut and and non-processed blot image of p-NF-κB and corresponding β-actin

Samples are labelled throughout as follows.

NG: normal group

MG: model group

BUDE-L: BUDE low dose group, 5 μM

BUDE-M:BUDE middle dose group, 15 μM

BUDE-H: BUDE high dose group, 25 μM

This is the result of WB experiment with 3 samples in each group. All proteins are the same batch of samples. All bands were incubated with the target protein first followed by eluted with stripping buffer and finally incubated with β-actin.

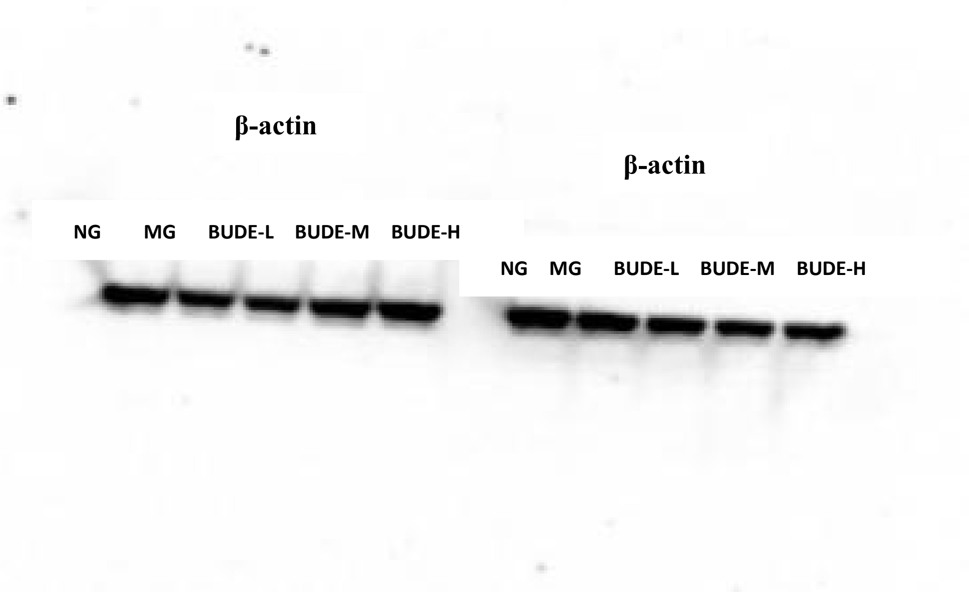

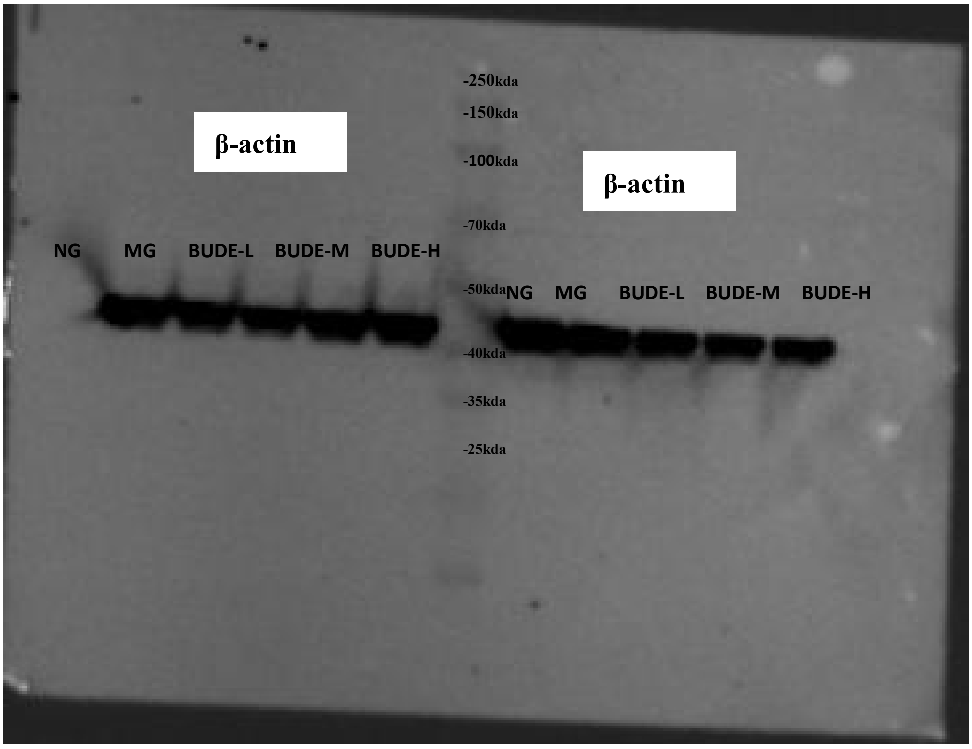

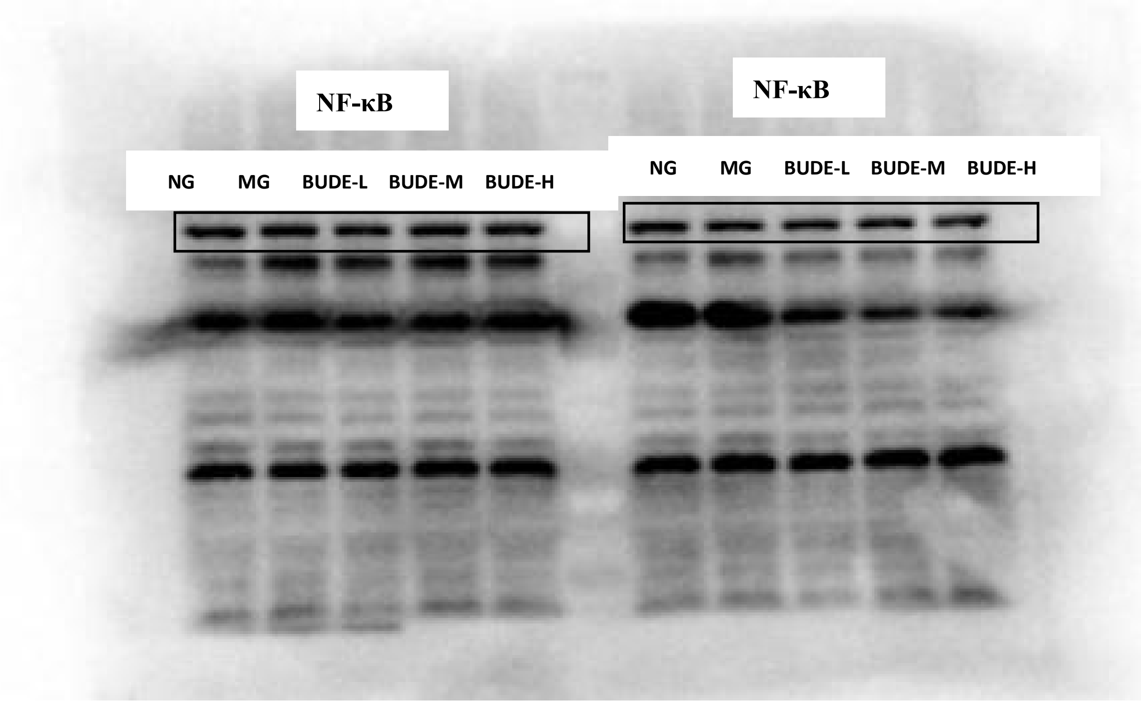
NF-κB
NF-κB
NG MG BUDE-L BUDE-M BUDE-H
NG MG BUDE-L BUDE-M BUDE-H

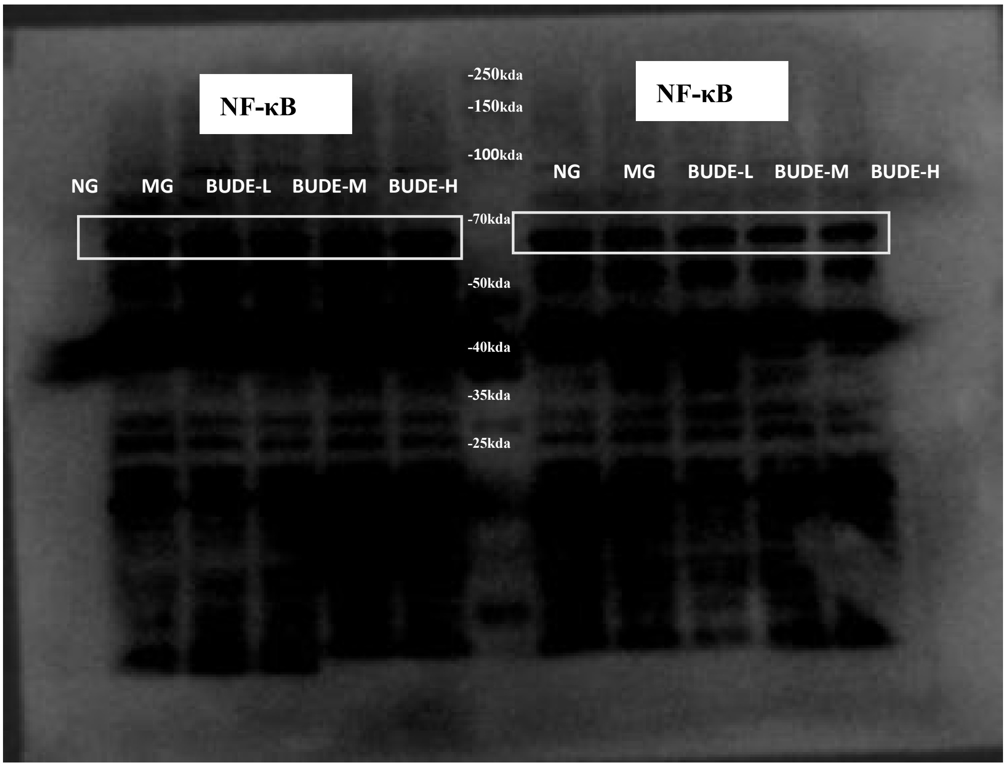
NF-κB
NF-κB
-250kda
-150kda
-100kda
-70kda
-50kda
-40kda
-35kda
-25kda
NG MG BUDE-L BUDE-M BUDE-H
NG MG BUDE-L BUDE-M BUDE-H

β-actin

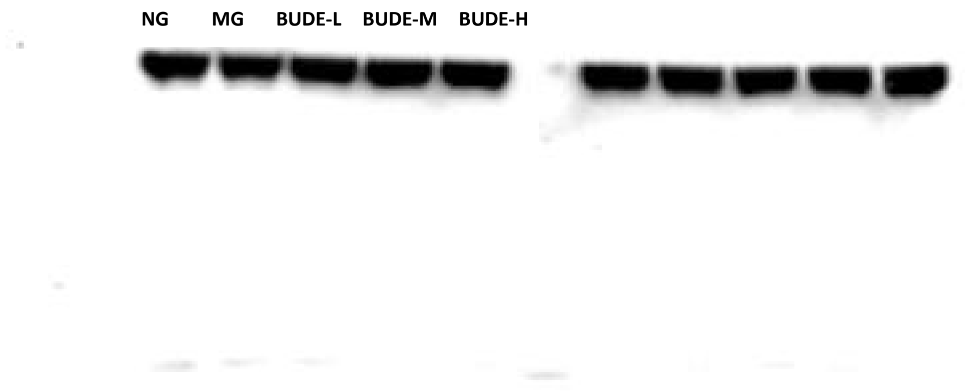
NG
MG
BUDE-L
BUDE-M
BUDE-H

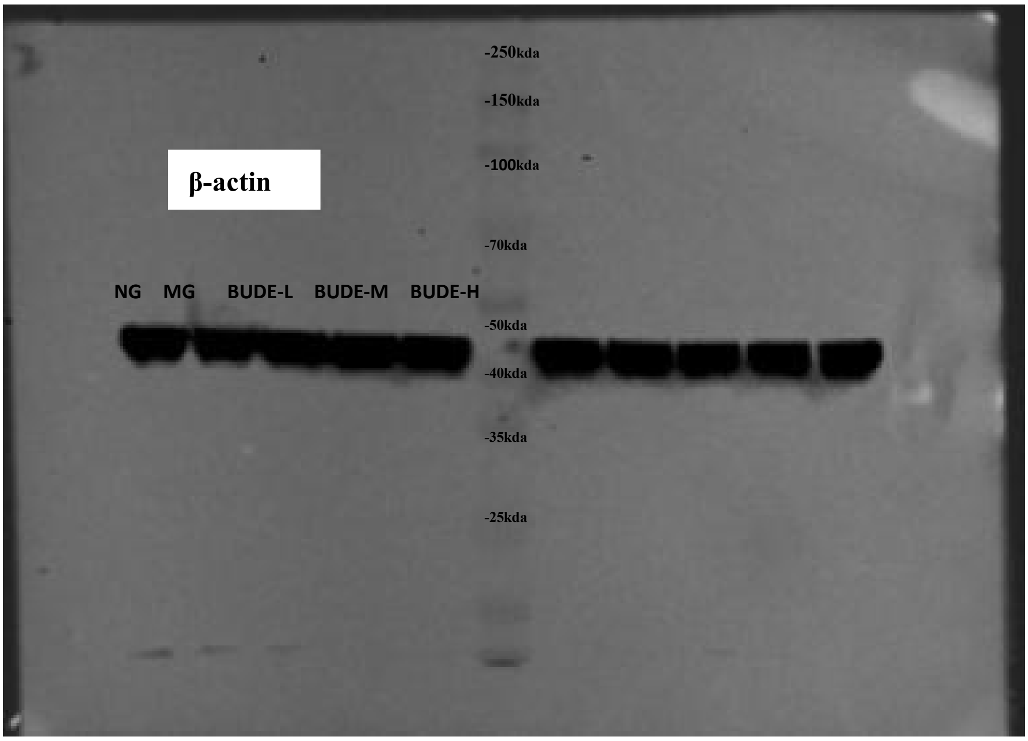
β-actin
NG
MG
BUDE-L
BUDE-M
BUDE-H
-250kda
-150kda
-100kda
-70kda
-50kda
-40kda
-35kda
-25kda

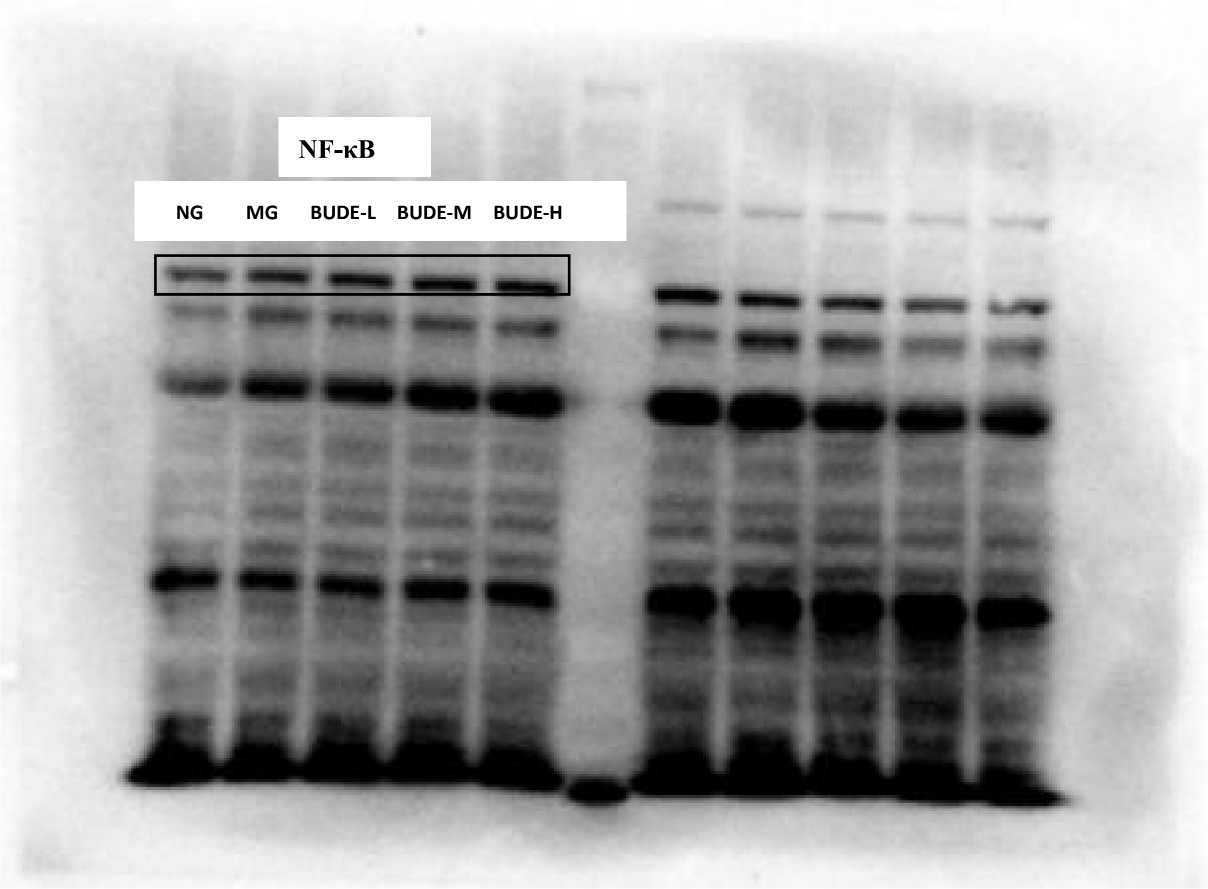
NF-κB
NG MG BUDE-L BUDE-M BUDE-H

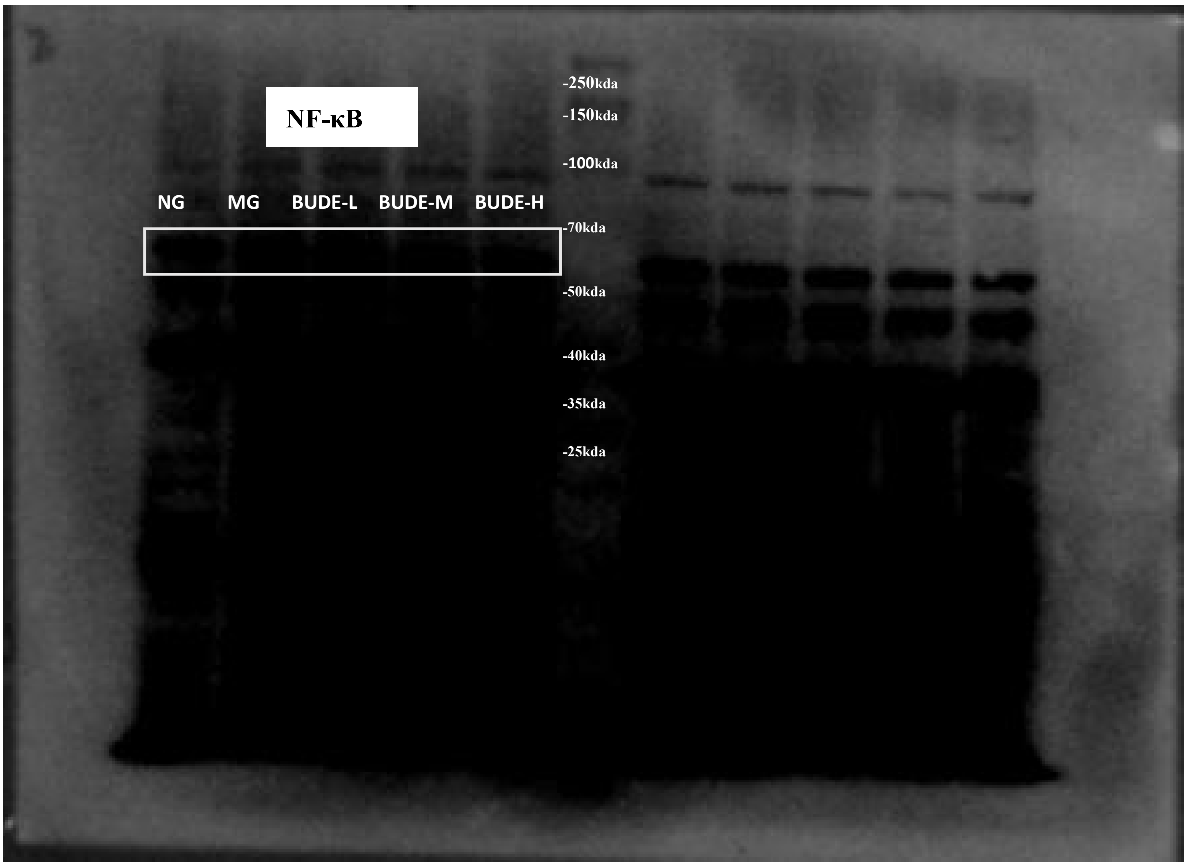
NF-κB
NG MG BUDE-L BUDE-M BUDE-H
-250kda
-150kda
-100kda
-70kda
-50kda
-40kda
-35kda
-25kda

Fig.2 The uncut and and non-processed blot image of NF-κB and corresponding β-actin
Samples are labelled throughout as follows.

NG: normal group

MG: model group

BUDE-L: BUDE low dose group, 5 μM

BUDE-M:BUDE middle dose group, 15 μM

BUDE-H: BUDE high dose group, 25 μM

This is the result of WB experiment with 3 samples in each group. All proteins are the same batch of samples. All bands were incubated with the target protein first followed by eluted with stripping buffer and finally incubated with β-actin.
